# Combining Dietary Intervention with Metformin Treatment Enhances Non-Alcoholic Steatohepatitis Remission in Mice Fed a High-Fat High-Sucrose Diet

**DOI:** 10.3390/biom12121787

**Published:** 2022-11-30

**Authors:** Gerard Baiges-Gaya, Elisabet Rodríguez-Tomàs, Helena Castañé, Andrea Jiménez-Franco, Núria Amigó, Jordi Camps, Jorge Joven

**Affiliations:** 1 Department of Medicine and Surgery, Rovira i Virgili University (URV), 43201 Reus, Spain; 2 Unitat de Recerca Biomèdica (URB-CRB), Hospital Universitari de Sant Joan, Institut d’Investigació Santiària Pere i Virgili (IISPV), 43201 Reus, Spain; 3 CIBER of Diabetes and Associated Metabolic Disease (CIBERDEM), ISCIII, 28029 Madrid, Spain; 4 Biosfer Teslab, 43201 Reus, Spain; 5 Campus of International Excellence Southern Catalonia, 43003 Tarragona, Spain

**Keywords:** obesity, NAFLD, liver, adipose tissue, lipidomic, mice

## Abstract

Non-alcoholic fatty liver disease (NAFLD) and non-alcoholic steatohepatitis (NASH) are serious health concerns for which lifestyle interventions are the only effective first-line treatment. Dietary interventions are effective in body weight reduction, but not in improving insulin sensitivity and hepatic lipid mobilization. Conversely, metformin increases insulin sensitivity and promotes the inhibition of de novo hepatic lipogenesis. In this study, we evaluated the metformin effectiveness in NASH prevention and treatment, when combined with dietary intervention in male mice fed a high-fat high-sucrose diet (HFHSD). Eighty 5-week-old C57BL/6J male mice were fed a chow or HFHSD diet and sacrificed at 20 or 40 weeks. The HFHSD-fed mice developed NASH after 20 weeks. Lipoprotein and lipidomic analyses showed that the changes associated with diet were not prevented by metformin administration. HFHSD-fed mice subject to dietary intervention combined with metformin showed a 19.6% body weight reduction compared to 9.8% in those mice subjected to dietary intervention alone. Lower hepatic steatosis scores were induced. We conclude that metformin should not be considered a preventive option for NAFLD, but it is effective in the treatment of this disorder when combined with dietary intervention.

## 1. Introduction

Obesity is a metabolic disease that increases the risk of developing non-alcoholic fatty liver disease (NAFLD). The onset of NAFLD consists of an increase in the lipid content of the liver and, over time, alterations leading to non-alcoholic steatohepatitis (NASH), which is characterized by hepatocyte death, inflammation, and fibrosis. Lipid metabolism requires complex regulations to maintain plasma membrane fluidity and lipoprotein synthesis needed for cellular viability [1,2,3]. Under normal conditions, excessive intracellular lipid levels are metabolized, transported in secreted lipoproteins, or stored as lipid droplets. However, in NAFLD, the lipoprotein secretion and glycerophospholipid synthesis by the endoplasmic reticulum may be impaired and lead to alterations in very-low-density lipoprotein (VLDL) size and glycerophospholipid profile [4,5,6,7]. In this metabolic context, lipid mobilization deteriorates, causing the accumulation of cholesterol esters in the hepatocyte, leading to NASH development [8,9,10]. The lack of effective pharmacological strategies makes the management of NAFLD difficult and becomes an important health challenge worldwide. Metformin is the first-line drug in type 2 diabetes mellitus management: a function of increasing insulin sensitivity. However, there is insufficient evidence on the role of metformin within the spectrum of metabolic derangements of NAFLD. Earlier animal studies suggested that metformin is effective in reducing hepatic steatosis by inhibiting de novo hepatic lipogenesis and, as such, preventing the onset and progression of NAFLD. However, most of these studies have been conducted in animals fed a high-fat diet (HFD) [11,12,13] and the results from our group [14] and others [15] have reported that, compared to HFD-fed mice, high-fat high-sucrose diets (HFHSD) promote a phenotype characterized by higher hepatic steatosis scores, oxidative stress, and inflammatory environment, together with alterations in autophagy. The results suggest that such a diet could be a better mouse model for the study of NASH. Caloric restriction is effective in body weight reduction, while amelioration of the hepatic fat content [16] is less effective in increasing insulin sensitivity and adipose tissue lipid mobilization [17] Thus, the present study addressed the effects of caloric restriction and metformin treatment in the context of severe obesity and NASH; the outcome having clinical relevance in the treatment of morbid obesity. Using a lipidomic approach, the objectives of the study were to evaluate (1) if metformin treatment protects against NASH development in HFHSD-fed mice; and (2) the possible combined effects of dietary intervention and metformin administration in NAFLD resolution.

## 2. Materials and Methods

### 2.1. Mice Study Protocol

Four-week-old C57BL/6J mice were obtained from ENVIGO (Barcelona, Spain) and were housed 4 per cage in a temperature-controlled room (22 ± 2 °C) with a 12 h light/dark cycle, with “lights on” corresponding to 8 am. All mice were male, acclimatized to the animal house for 1 week. They were fed a diet (TD.2018, Teklad, Harlan Laboratories Inc., Madison, WI, USA) composed of 58% carbohydrates, 24% protein, and 18% fat. During the study, mice were fed with a chow diet (TD.2014, Teklad, Harlan Laboratories Inc., Madison, WI, USA), composed of 67% carbohydrates, 20% protein, and 13% fat, or an HF-HSD (TD.08811, Teklad, Harlan Laboratories Inc., Madison, WI, USA) containing 40.7% carbohydrates (29% sucrose), 14.7% protein and 44.6% fat (Table S1). Mice were monitored daily for well-being and signs of discomfort and distress. Body weight was monitored weekly, and food and water intakes were measured daily. Metformin was administrated in drinking water daily (300 mg/kg body weight/day). Animal experiments were approved by the Animal Ethics Committee of Universitat Rovira i Virgili and authorized by the Directorate General of Environmental Policies and the Natural Environment of the Government of Catalonia (reference number: 10281). 

### 2.2. Mice Study Design

The study was carried out on 80 male mice. Five-week-old animals were randomly divided into 6 experimental groups. Sixteen mice were fed CD and were sacrificed by cervical dislocation under general anesthesia in groups of 8 at 25 and 45 weeks of age. The same design was repeated for animals fed CD + metformin, HFHSD alone, and HFHSD + metformin. The remaining 16 animals received HFHSD or HFHSD + metformin. Halfway through the study, the regimen was switched from HFHSD to CD (termed HFHSD-CD and HFHSD + metformin-CD + metformin), reducing daily food intake by 3.5 kcal. The animals were sacrificed at 45 weeks of age. Tissue collection was at 8 am (Zeitgeber time 0). See Figure S1 for experimental design.

### 2.3. Glucose Tolerance Test

Two weeks before scheduled sacrifice, the mice were fasted for 14 h (from 6 pm to 8 am) and were injected with a glucose solution (2 g glucose per kg body weight, i.p.). Blood samples for glucose measurement were taken at 15, 30, 60, and 120 min post-injection, and glucose was measured using a handheld glucometer (Accu-Check ^®^ glucose reader, Roche Diagnostics, Basel, Switzerland).

### 2.4. Standard Biochemical Analyses

Serum glucose, cholesterol, and triglycerides concentrations, and alanine aminotransferase activities were measured in a Roche Modular P800 Chemistry Analyzer (Roche Diagnostics).

### 2.5. Histological Analysis

Liver and adipose tissue samples, at the conclusion of each dietary period, were removed and fixed in formalin and embedded in paraffin for hematoxylin/eosin staining. The degree of hepatic impairment was estimated using the NAFLD activity score (NAS score). This scoring system is based on histological features classified into three categories: steatosis (graded from 0 to 3), lobular inflammation (graded from 0 to 2), and hepatocellular ballooning (graded from 0 to 2) [18]. Samples were assessed by an experienced pathologist of the team (J.J.) who was blinded with respect to the provenance of the samples. NASH was considered when the NAS score was ≥5. The average adipocyte size from visceral and subcutaneous adipose tissue was estimated using the Image J 1.51 software (National Institutes of Health, Bethesda, MD, USA) with the macro MRI’s adipocyte tool. Sections were assessed using optical microscopy (Eclipse E600, Nikon, Tokyo, Japan), and images were recorded using NIS-Elements F 4.00.00 software (Nikon, Tokyo, Japan).

### 2.6. Lipoprotein Analyses by Nuclear Magnetic Resonance (^1^H-NMR) Spectroscopy

The lipoprotein profiles of serum samples were analyzed by ^1^H-NMR (Liposcale^®^, Biosfer Teslab, Reus, Spain) as previously described [5].

### 2.7. Isolation of Mature Adipocytes

Visceral (epididymal and retroperitoneal) and subcutaneous (inguinal and anterior) adipose tissues were removed post-sacrifice and resuspended in a digestion solution containing 10 mL of DMEM/F12 and 10 mg of collagenase type II (C6885, Sigma Aldrich, St.Louis, MO, USA) for 50 min at 37 °C with agitation at 100 rpm. Digested samples were then passed through a 250 µm cell strainer (S1020, Sigma Aldrich, St. Louis, MO, USA) into fresh sample tubes and centrifuged at room temperature at 300× *g* for 5 min to separate adipocytes from the stromal vascular fraction. Adipocytes were separated by pipetting, washed in phosphate-buffered saline solution, and used directly for lipid analysis.

### 2.8. Semi-Targeted Lipidomics

Apolar lipids were extracted from liver and adipocytes with methanol, and polar lipids with methanol and chloroform, as described in detail [14,19]. Lipid samples were analyzed by liquid chromatography coupled to time-of-flight mass spectrometry. We detected 292 and 190 lipid species from liver and adipocytes respectively, The following categories were identified: Fatty acyls (FAs) (fatty acids (FA), acyl-carnitines (CAR), hydroxy fatty acids (HFA), N-acyl-ethanolamine (NAE)); glycerolipids (GLs) (triglycerides (TG), diglycerides (DG)); glycerophospholipids (GPs) (ester-linked phosphatidylcholines (PC), ester-linked lysophosphatidylcholines (LPC), ester-linked lysophosphatidylethanolamines (LPE)); sphingophospholipids (SPs) (sphingomyelins (SM)); and sterol lipids (ST) (bile acids (BAs), cholesterol ester (CE), steroid hormones). Data are expressed as internal standard response ratios.

### 2.9. Statistical Analysis

All data from box plots are represented as means, with bars showing the minimum and maximum values. Error bars of bar plots represent standard deviation values. Some data are represented in lollipop and volcano plots. Statistical significance of quantitative variables of lipid species was assessed using Wilcoxon rank-sum test. A *p* < 0.05 was considered statistically significant. Dimensionality reduction techniques (scikit-learn package) [20], lollipop (matplotlib package) [21], and volcano plots (bioinfokit package) were constructed using the Jupyter Notebook via the Anaconda environment [22] and written in Python code. Finally, GraphPad Prism version 9.0.1 (San Diego, CA, USA) was used to generate box and bar plots. Linear discriminant analysis (LDA) was used as a supervised technique method for dimensionality reduction analysis. LDA projects the data onto a low-dimensional space and is useful in exploring differences between groups. 

## 3. Results

### 3.1. Metformin Does Not Prevent HFHSD-Associated NAFLD Development and Metabolic Derangements

**Figure 1:**
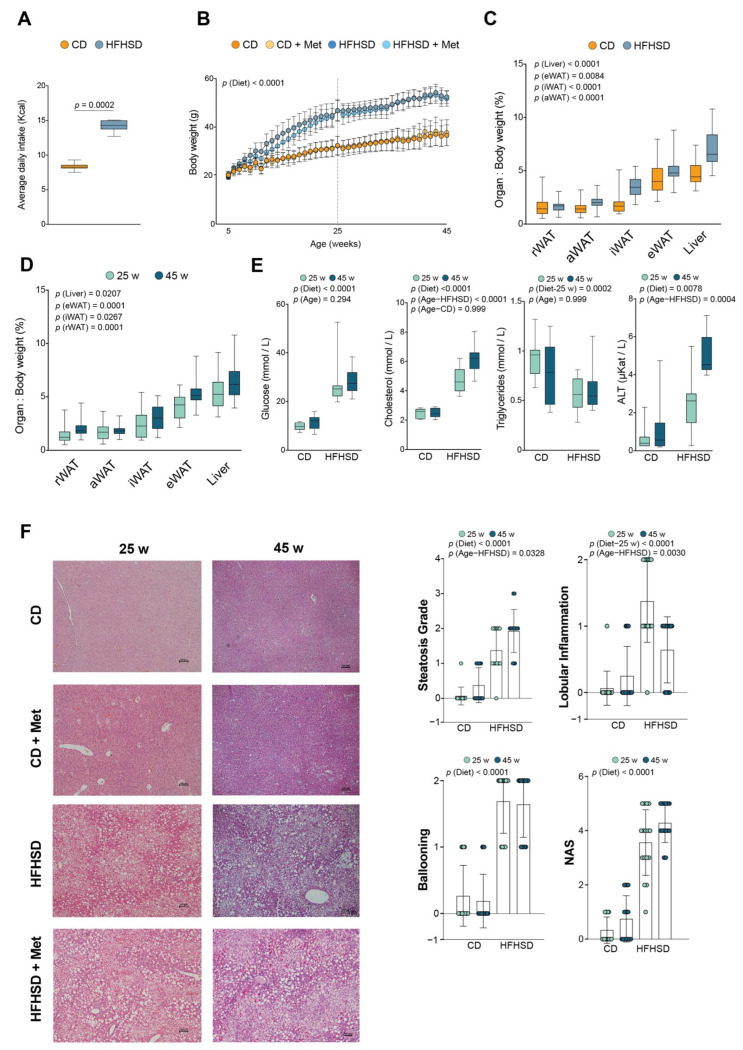
(**A**) Caloric intake and (**B**) body weight gain curves of CD- and HFHSD-fed mice (*n* = 8/group); (**C**,**D**) Tissue weights in relation to dietary treatment and age; (**E**) Plasma glucose, cholesterol, triglycerides, and alanine aminotransferase activity (ALT) in animals sacrificed at 25 and 45 weeks of age (*n* = 8/group); (**F**) Representative histological images of liver sections of mice (*n* = 8/group) according to the type of diet, age, and metformin administration (on the left), and scores of hepatic steatosis, lobular inflammation, ballooning, and NAFLD activity score (on the right). Scatter and bar plots are presented as a means and SD, while box plots are shown as means, maximum and minimum. *p* values < 0.05 are considered significant. (Wilcoxon-rank sum test). aWAT: anterior white adipose tissue; CD: chow diet; eWAT: epididymal white adipose tissue; HFHSD: high-fat high sucrose diet; iWAT: inguinal white adipose tissue; Met: metformin; rWAT: retroperitoneal white adipose tissue; w: weeks. In panels (**A**,**C**–**F**) (right), mice with and without metformin were grouped because metformin administration did not produce any changes in the analyzed variables.

The average daily caloric intake in HFHSD-fed mice was almost double that in the CD-fed animals (Figure 1A). 

Body weight increased significantly and independently of metformin administration (Figure 1B). We observed that diet and age contributed to the increase in tissue weight (Figure 1C,D). Mice fed on an HFHSD developed a phenotype characterized by higher plasma glucose and cholesterol, and lower triglyceride concentrations together with higher alanine aminotransferase activities than mice fed on CD (Figure 1E). In CD-fed mice, metformin administration slightly increased the cholesterol concentration in 45-week-old mice, whereas in HFHSD-fed mice with metformin, cholesterol and triglyceride concentrations were decreased (Figure S2). In addition, metformin improved glucose tolerance in 25-week-old mice, but not in 45-week-old mice (Figure S3A,B). We found that cholesterol levels and alanine aminotransferase activities increased with age in HFHSD-fed mice (Figure 1E). 

In mice receiving HFHSD, the total number of lipoprotein particles was higher than in those given CD, with an increase in the number of low-density lipoprotein particles (LDL-p) and decreases in very low-density and high-density lipoprotein particles (VLDL-p) and (HDL-p), respectively (Figure S4A). Medium-sized VLDL-p and large HDL-p increased in HFHSD-fed mice; a phenomenon that is accentuated with age (Figure S4B). Total lipoprotein cholesterol was higher in mice fed HFHSD, whereas the total lipoprotein triglycerides were higher in mice fed CD. We found that the LDL-p population in HFHSF-fed mice was enriched with cholesterol and triglycerides, and these alterations were exacerbated with age (Figure S4C,D). In addition, we observed that metformin slightly decreased the VLDL-p as well as the TG and cholesterol content linked to VLDL in 25-week-old mice fed CD. In 45-week-old mice, fed HFHSD, slightly decreased VLDL-p and increased TG content linked to IDL were observed (Figure S5). Further, we observed that 25-week-old mice fed CD + metformin had smaller VLDL-p than mice not receiving metformin (Figure S6A). In summary: age worsened the HFHSD-associated lipoprotein profile, while metformin administration did not ameliorate it.

Hepatic histological analysis showed that dietary treatment was the main cause of NAFLD development, increase in hepatic steatosis, lobular inflammation, and ballooning scores. As a result, HFHSD-fed mice exhibited a phenotype associated with the diagnosis of probable NASH (NAS = 3–4) and definite NASH (NAS ≥ 5), independently of metformin administration Figure 1F and Figure S7). Moreover, the hepatic steatosis scores tended to increase with age.

### 3.2. NAFLD Was Associated with Alterations in Cholesterol-Related Lipids and Glycerophospholipids 

**Figure 2:**
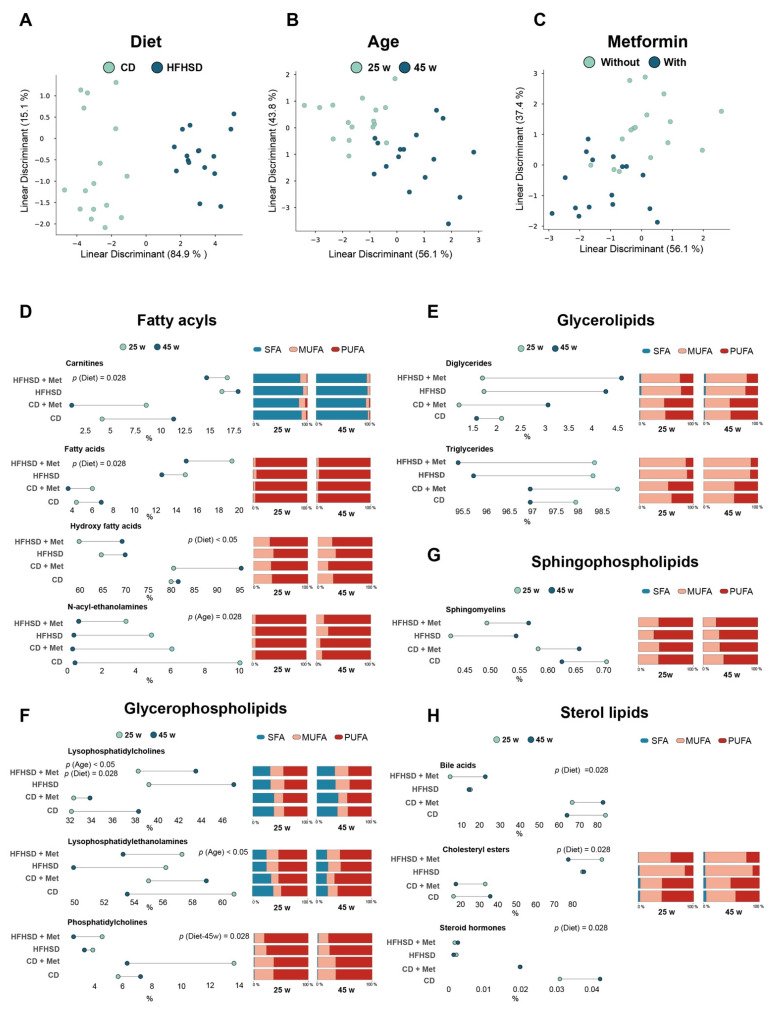
Hepatic lipid remodeling in mice, segregated by diet, age, and metformin: (**A**–**C**) Linear discriminant analysis (LDA) showing the lipid signature of liver tissue according to dietary type, age, and metformin administration; (**D**–**H**) Lollipop plots showing the age of mice in sky-blue dots (25 w) and blue dots (45 w). Horizontal axis displays the mean relative abundance of lipids, while the vertical axis reflects the group of mice (*n* = 4/group) *p* values < 0.05 are considered significant. (Wilcoxon-rank sum test). CD: chow diet; HFHSD: high-fat high-sucrose diet; Met: metformin; MUFA: monounsaturated fatty acids; PUFA: polyunsaturated fatty acids; SFA: saturated fatty acids; w: weeks.

LDA showed that overall hepatic lipidome was altered mainly by the dietary treatment, while age and metformin played secondary roles (Figure 2A–C and Figure S8A–C). 

As such, hepatic GLs were the most abundant lipid category in CD-fed mice, followed by GPs, FAs, SPs, and ST. HFHSD-fed animals had higher levels of ST than SPs. Despite the importance of diet in modifying the lipid category relative abundance, we also found alterations in the lipid composition of FAs, GPs, and ST, i.e., the FAs pool in mice with NAFLD was characterized by a decrease in hydroxy fatty acids, an oxylipin type, and increase in CAR and FA (Figure 2D). In the GPs pool, the levels of PC decreased, whereas the levels of LPC increased, suggesting that phospholipid hydrolysis is increased (Figure 2F). Finally, in the ST pool, we found that free cholesterol was mainly depleted in forming CE and, therefore, the concentrations of BA and St decreased (Figure 2H). In parallel with these findings, we observed that DG, CE, TG, LPC, and LPE increased the monounsaturated fatty acid (MUFA) content in NAFLD mice, whereas the polyunsaturated fatty acids (PUFA) content linked to PC decreased (Figure 2E–H). With respect to age contribution to hepatic lipid remodeling, we found that NAE and LPE decreased with age, while the LPC species increased, independently of diet and metformin administration (Figure 2A,F). We did not find any alterations associated with metformin.

### 3.3. Age Determined the Lipid Remodeling in Adipocytes

**Figure 3:**
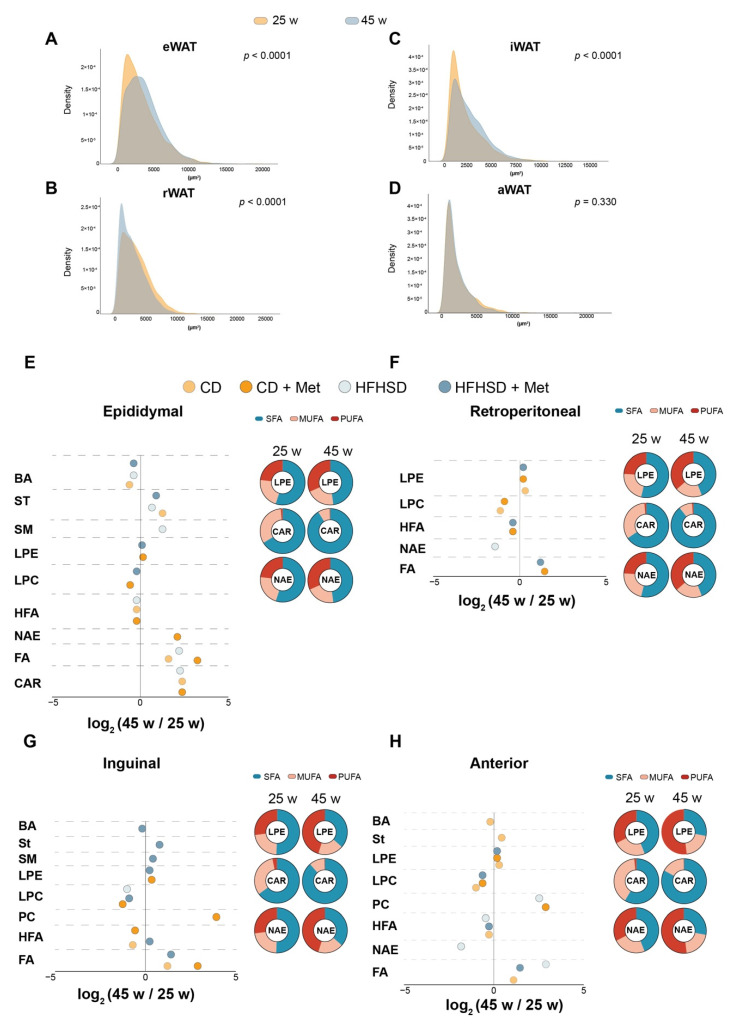
Adipocyte lipid signature segregated according to age. The density plots show the relative frequency of histological adipocyte size: (**A**) epididymal; (**B**) retroperitoneal; (**C**) inguinal; (**D**) anterior white adipose tissues. The age of mice depicted by colored dots; sky-orange (25-week-old) and sky-blue (45-week-old). Representation of significant lipid classes, showing mean log2 fold change (45-week-old mice/25-week-old mice) and the changes in the degree of unsaturation of lipid; (**E**) epididymal (top left); (**F**) retroperitoneal (top right); (**G**) inguinal (bottom left); (**H**) anterior (bottom right) adipocytes. Each group of mice (*n* = 4/group) represented by colored dots: sky-orange (CD-fed mice); orange (CD-fed mice + metformin); sky-blue (HFHSD-fed mice); blue (HFHSD-fed mice + metformin). *p* values < 0.05 are considered significant. (Wilcoxon-rank sum test). BA: bile acids; CAR: carnitines; CD: chow diet; HFHSD: high-fat high-sucrose diet; DG: diglycerides; FA: fatty acids; HFA: hydroxy fatty acids; LPC: lysophosphatidylcholines; LPE: lysophosphatidylethanolamines; Met: metformin; MUFA: monounsaturated fatty acids; NAE: N-acyl-ethanolamine’s; PC: phosphatidylcholine; PUFA: polyunsaturated fatty acids; SFA: saturated fatty acids; SM: sphingomyelins; St: steroid hormones; w: weeks.

To ascertain whether metformin affected the lipid signature of adipocytes, we analyzed the histological adipocyte size and lipid alterations from visceral (epididymal and retroperitoneal) and subcutaneous (inguinal and anterior) adipocytes in 25-week and 45-week-old mice. Our data showed that epididymal adipocyte size increased with age, HFHSD, and metformin, whereas retroperitoneal adipocyte size decreased with age and HFHSD. Inguinal subcutaneous depots increased with age and HFHSD while being decreased in metformin-treated mice. Finally, anterior subcutaneous adipocyte size decreased in HFHSD-fed mice (Figure 3A–D, Figures S9 and S10A–C). 

White adipose tissue acts as the main lipid storage facility by incorporating TG and DG into the adipocytes. Thus, adipocyte lipidome in CD-fed mice revealed that GLs were the main lipid category in visceral as well as subcutaneous depots in 25-week-old mice. The FAs, GPs, SPs, and ST were the most important lipid categories in epididymal and inguinal regions, whereas, in retroperitoneal and anterior adipocytes, the categories were SPs, FAs, GPs, and ST, suggesting that the adipocyte lipid signature is specific to the anatomical region (Figures S11–S14).

When 25-week-old CD-fed mice received metformin, we observed a remodeling of the lipid signature in epididymal, retroperitoneal, and anterior adipocyte depots in epididymal regions. We observed an increase in TG and a decrease in HFA, LPE, and LPC species, while in retroperitoneal adipocytes, HFA was increased. Finally, in anterior adipocytes, we observed a decrease in TG and an increase in SM compared to mice without metformin (Figures S11–S14). However, the metformin effects on HFSHD-fed 25-week-old mice were less relevant, with increases only in SM in epididymal adipocytes being observed (Figure S8E). 

These alterations in CD-fed mice contributed to the lipid class weight redistribution within each lipid category. Thus, we observed an increase in fatty acids in the FAs pool of epididymal adipocytes, and an increase in DG in the GLs pool of anterior adipocytes (Figure S9E–G).

With respect to the effects of HFHSD, we observed that the increase in NAE levels in visceral depots and the increase in PC species in retroperitoneal adipocytes were independent of metformin administration. Moreover, in mice treated with metformin, we found specific alterations characterized by the increase in LPE species in epididymal, and a decrease in retroperitoneal, regions. Finally, we observed that mice without metformin had decreased levels of steroid hormones in retroperitoneal adipocytes (Figure S10E,F).

In subcutaneous depots, we found that inguinal adipocytes increased the bile acid concentrations while the steroid hormones decreased in mice with and those without metformin; the decrease in the SM species was specific to metformin treatment. Finally, in anterior adipocytes, we observed a decrease in SM in metformin-treated mice and a decrease in FA in mice without metformin (Figure S10G,H). Our lipidomic data also revealed the contribution of diet to qualitatively modifying the lipid aspects; i.e., we found that both visceral and subcutaneous depots increased the MUFA content in TG, DG, FA, and LPC species, independent of metformin administration (Figure S10E–H).

Of note, we found that age acts as the main factor in remodeling the lipid signature of adipocytes (Figure 3E–H and Figure S11–S14). We observed that all the 45-week-old groups of mice exhibited the same signature in both visceral and subcutaneous adipocytes, independent of diet and metformin administration; i.e., the adipocytes developed a phenotype associated with reduced lipid mobilization and favored lipid storage as triglycerides and diglycerides. Moreover, we found that GPs levels, which are related to plasma membrane lipids, decreased (Figure S15). These alterations induced changes in the lipid class distribution, increasing the importance of steroid hormones within the ST pool, as well as the FA in the pool of FAs, due to the decrease in NAE levels. The GP pools were characterized by decreased LPC species and increased LPE species; subcutaneous depots also increased the PC species (Figure 3E–H). Finally, we observed that age contributes to an increase in the saturated fatty acid content in CAR, and the PUFA content in LPE and NAE (Figure 3D–H).

In summary, metformin administration can modify the adipocyte lipid signature in young mice fed on CD, whereas these changes are reduced when HFHSD is administrated, or completely lost as age advances.

### 3.4. Combining Dietary Intervention with Metformin Treatment Promotes Hepatic Steatosis Remission

**Figure 4:**
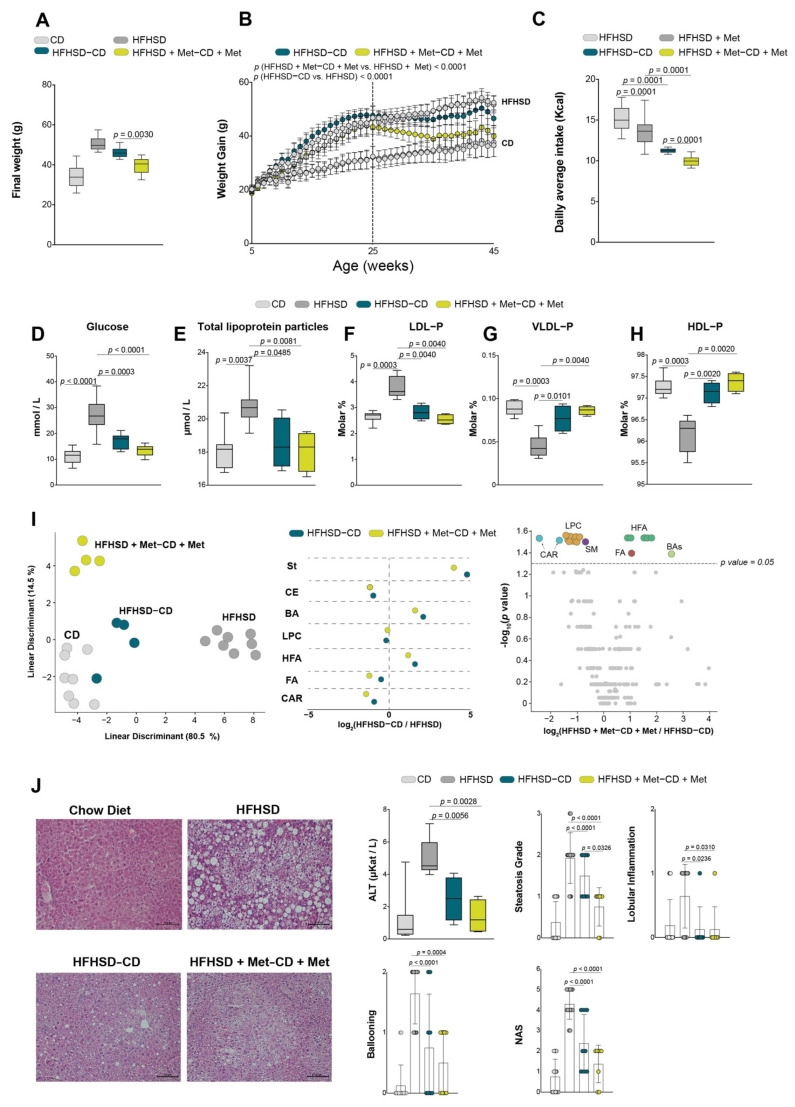
Combined effects of dietary intervention and metformin in the NAFLD remission: (**A**) final body weight; (**B**) body weight curve; (**C**) caloric intake (*n* = 8/group). The concentration of (**D**) glucose (*n* = 8/group); (**E**) total lipoprotein parameters (*n* = 4/group); (**F**–**H**) VLDL-p, LDL-p, and HDL-p molar abundance (expressed as a percentage of total measured lipoprotein particles) (*n* = 4/group); (**I**) lipid signature analysis and significant alterations in liver comparing the effects of calorie restriction with or without metformin versus obese mice lipidome (*n* = 4/group); (**J**) representative histology images of liver sections with the NAFLD activity score, and alanine aminotransferase activity (*n* = 8/group). Scatter and bar plots are presented as means and SD, while box plots are shown as means, maximum and minimum. *p* values < 0.05 are considered significant. (Wilcoxon-rank sum test). ALT: alanine aminotransferase; BA: bile acids; CAR: carnitines; CD: chow diet; CE: cholesterol esters; FA: fatty acids; HDL: high-density lipoproteins; HFA: hydroxy fatty acids; HFHSD: high-fat high-sucrose diet; LDL: low-density lipoproteins; LPC: lysophosphatidylcholines; Met: metformin; NAS: NAFLD activity score; p: particles; SM: sphingomyelins; St: steroid hormones; VLDL: very low-density lipoproteins.

Swapping from the HFHSD diet to the CD diet produced a caloric restriction of 3.48 kcal/day (from 14.72 to 11.24 kcal/day) and swapping from HFHSD + metformin to CD + metformin produced similar results (3.55 kcal/day; from 13.56 to 10.01 kcal/day). Body weight was significantly reduced under caloric restriction, with the largest reduction occurring when the diet switch was combined with metformin treatment (Figure 4A,B).

Glucose, total lipoprotein particles, and lipoprotein profile improved in mice subjected to dietary intervention compared to mice fed on an HFHSD, independent of metformin treatment, and without any improvement in the glucose tolerance test (Figure 4D–H and Figure S3C). Further, the hepatic lipidome returned to normal when mice were switched from HFHSD to CD, independent of metformin administration. Thus, cholesterol metabolism was ameliorated, resulting in a decrease in CE and an increase in BAs and steroid hormone levels. Additionally, we found that the CAR and FA levels decreased, while the HFA increased. 

Liver histology revealed less hepatic steatosis in mice subjected to dietary intervention + metformin than in animals treated with dietary intervention alone. Alanine aminotransferase activity, lobular inflammation, and ballooning improved compared to HFSHD, independent of metformin treatment (Figure 4I). The lipid signature of adipocytes from visceral and subcutaneous adipose tissue also improved (Figure S16A–G). In all adipocytes, we observed an increase in PUFA-containing TG and DG, as well as an increase in PUFA-containing LPC in both visceral depots (Figure S16B–H). We did not observe relevant alterations relating to metformin treatment.

## 4. Discussion

We did not find any protective effect of metformin alone in the liver histology after 20 and 40 weeks of dietary treatment (25 and 45 weeks of age); i.e., the observations of changes in biochemical parameters, lipoprotein particles, weight gain, and hepatic lipidome in mice fed HFHSD and metformin were like those of mice fed with HFHSD alone. Unlike other reports, these data do not support the use of metformin as a therapeutic strategy to prevent NAFLD in obese mice. Previous studies in ob/ob mice treated with metformin for 4 weeks showed weight reduction and hepatic steatosis prevention [23,24], but these experiments had been performed in animals fed on a CD diet. As such, the effect of metformin administered in this model, with a hypercaloric diet, had not been explored. Despite the controversial effect of metformin in in vivo studies, some human studies suggested that metformin maintains the intrahepatic TG content [25,26,27]. 

When lipids reach the liver, the free fatty acids are esterified into CE and TG by acyl-CoA cholesterol acyltransferase and di-acylglycerol-O-acyltransferases, respectively, and incorporated into the hepatic lipid droplets. The glycerophospholipid metabolism plays a crucial role in the biogenesis of lipid droplets, which are mainly composed of PC. However, when PC levels decrease, lipid droplet stability can be compromised, resulting in larger droplets. In this sense, we observed that mice fed on an HFHSD, independent of metformin administration, showed a hepatic reduction in PC and a concomitant increase in LPC. The findings observed in HFHSD-fed mice could be explained, at least in part, by an increase in phospholipase activity that cleaves an acyl-chain of PC to be then converted in LPC. Indeed, the lower hepatic PC levels correlate with the presence of larger hepatic droplets. Therefore, according to our results, the decrease in the hepatic PC content might be an important aspect of NAFLD progression. Parallel to these results, we also observed an increase in hepatic fatty acids and acyl-carnitines in mice fed on an HFHSD. In patients with NAFLD, a decrease in acyl-carnitine transport to the inner mitochondrial membrane for B-oxidation results in hepatic fatty acid accumulation [28,29,30]. Excess saturated fatty acids and free cholesterol can promote hepatic lipotoxicity. Free cholesterol is rapidly metabolized to synthesize BA and steroid hormones or esterified into CE by incorporating saturated or monounsaturated, very long-chain fatty acids. Our lipidomic results suggest that cholesterol metabolism changes as liver disease advances. Thus, mice fed on an HFHSD and independently of metformin treatment exhibited higher levels of hepatic CE and lower levels of BA and steroid hormones than CD-fed mice. Recent evidence suggests that CE accumulation is associated with NAFLD progression. For example, patients with hepatocellular carcinoma showed higher plasma concentrations of CE than patients with cirrhosis [31,32]. Indeed, ACAT2-/- (also abbreviated as SOAT2) mice had improved insulin sensitivity, decreased lipid storage, and increased lipid oxidation pathways [33].

We have observed that the plasma concentrations of triglycerides in HFHSD mice are lower than those of CD. These results agree with the proposal that the normal liver would act as a fat reserve, protecting the rest of the organs when there is a strong nutrient overload. Then, fat overload would be associated with reduced VLDL secretion [34]. Studies in mice of acute overfeeding demonstrate a transient steatosis, which may represent a mechanism to deal with large influxes of nutrients. It has been suggested that the accumulation of large quantities of lipids in NAFLD may represent a maladaptation of physiologic systems in the liver designed to buffer short-term changes in nutritional status [35].

While we did not observe any preventive effect in the liver with respect to metformin treatment, we found that the adipocyte size in eWAT (visceral) and aWAT (subcutaneous) had higher and lower sizes, respectively, than in HFHSD-fed mice alone. CD, and metformin treatment of 25-week-old mice, exhibited lower levels of SM in anterior adipocytes than those mice without metformin. Further, 25-week-old mice receiving HFHSD + metformin had lower levels of SM in subcutaneous depots than metformin-treated mice in the CD group, suggesting that metformin could mediate the sphingophospholipid metabolism. Indeed, a human study in women with polycystic ovary syndrome observed that metformin treatment decreases serum SM levels as well as oxidized lipids; although, the mechanisms were not fully explained [36]. 

In addition, our data showed that adipocytes from visceral and subcutaneous adipose tissue displayed lipid remodeling in HFHSD-fed mice, with a decrease in PUFA content in DG, TG, FA, and LPC. Indeed, an earlier study demonstrated that adipose tissue macrophages in obesity display higher PC turnover, resulting in an increase in the amount of saturated fatty acids in plasma membrane phospholipids [37]. Another finding of note was the decrease in glycerophospholipids and the increase in glycolipids with age, independent of the diet and metformin treatment. As has been described, the levels of phospholipids in blood, liver, and brain decrease with age, and have a higher degree of saturated fatty acids [38,39]. Therefore, the decrease in the content of GPs could be a hallmark of the aging process. For example, in 94-week-old mice, the PC, PE, and SM levels in kidney samples decreased in comparison to 14-week-old mice [40]. Moreover, 108-week-old mice showed GPs alterations in the heart and serum [41]. On the other side, appropriate levels of PC are necessary for TG storage in adipocytes. During the differentiation of 3T3-L1 fibroblasts into mature adipocytes, the expression of phosphatidylethanolamine N-methyltransferase (PEMT), is high [42]. This enzyme is involved in the synthesis of PC through PE tri-methylation. Of note, in vitro studies in PEMT-/- (an enzyme involved in the synthesis of PC through the PE trimethylation) adipocytes, demonstrate that the low level in PC causes a smaller-sized adipocyte and higher basal hydrolysis of triglycerides [43]. Despite PC being an important factor in adipocyte metabolism, we did not observe higher serum triglyceride concentrations nor decreased adipocyte size with age. The increase in age-related lipid accumulation in adipocytes may be due to DGAT activity, i.e., esterification of DG with FA in TG synthesis. Indeed, DGAT1 gene deletion in mice has been shown to promote longevity and body leanness, indicating that losses in lipid mobilization could be an indication of an organism’s aging [44]. Taken together, these data indicate that metformin, at least in a long-term study, cannot prevent the development of age- and HFHSD-associated metabolic derangements.

In contrast to our earlier hypotheses, our data strongly suggest that metformin, combined with dietary intervention, promotes greater beneficial NASH remission than dietary intervention alone. These effects could be conducted through either dietary intervention or caloric restriction promoted by regimen change. Metformin-treated mice showed a greater body weight reduction (19.6% vs. 9.8% weight loss) and rapid reversal of hepatic steatosis; i.e., experimental animal studies have indicated that the combination of dietary intervention with metformin can be more beneficial than either of these interventions alone [11,45]. The improvement in biochemical parameters, such as lipoprotein concentrations and profiles were not metformin specific, whereas the glucose tolerance test did not improve in either intervention. Indeed, a recent study showed that rats fed on a calorie-restricted cafeteria diet (30% caloric restriction) did not have decreased circulatory levels of glucose, triglycerides, LDL-cholesterol, and HDL-cholesterol compared to those rats fed on a cafeteria diet without caloric restriction [46]. Thus, these results suggest that the beneficial effects we observed in mice subjected to the dietary intervention were mediated by the diet change and not by caloric restriction per se.

Our findings demonstrate that dietary intervention modifies the lipid signature in the liver and adipocytes, independent of metformin administration. Our lipidomic results indicate that hepatic CE decreases, while the bile acids and steroid hormones increase (mimicking CD-fed mice), while adipocytes from visceral and subcutaneous depots increased PUFA-containing DG, TG, and LPC; i.e., the effects of dietary intervention in the liver are quantitative, but more qualitative in adipocytes. Caloric restriction per se does not modify the lipid composition, but the type of diet may determine the qualitative lipid aspects such that dietary sources enriched with PUFA modify the phospholipid plasma membrane composition, promoting better metabolic outcomes, than SFA-enriched diets [47,48,49].

In summary, we have shown that metformin cannot prevent hepatic steatosis and age-related lipid alterations. As such, its administration for the preventive treatment of NAFLD should be taken with caution [27]. Most NAFLD models described in the literature are based on HFD, and the period of treatment is shorter than in our study. However, our data indicate a summation of the effects of dietary intervention and metformin resulting in better weight reduction, and a better amelioration of hepatic steatosis than dietary intervention alone. Of further note is the age contribution to the lipid remodeling of adipocytes. For example, all 45-week-old mice groups exhibited similar lipid signatures independently of diet and metformin group assignment in the study. These remodeling processes were characterized by an increase in lipid storage (mainly triglycerides and diglycerides) and a decrease in glycerophospholipids, which are important regulators of adipocyte size, plasma membrane fluidity, and lipoprotein metabolism. Our results suggest that, during the aging process, the metabolic program changes from lipid mobilization to lipid storage resulting in alterations in the adipocyte plasma membrane. This scenario invites consideration of whether current therapies are appropriate, or whether a new treatment approach is necessary in older subjects with NAFLD. 

This study has several limitations. First, the number of mice in each study group is small. We have tried to minimize the effects of low sample size by using nonparametric tests in the statistical analyses. Perhaps the low sample size is the cause of some paradoxical results, such as, for example, that 25-week-old mice have more lobular inflammation and a similar level of ballooning than 45-week-old animals. It may also be the reason for the high variability of some biochemical parameters. Moreover, the dose of metformin was proportionally higher than that used in clinical practice in patients with diabetes. The reason was to ensure the effect of metformin in the mice, bearing in mind that our route of administration of metformin was the water bottle and not the oral gavage. Moreover, the need for a calorie-restricted HFHSD-fed mice group in the study makes it difficult to conclude that the beneficial effects of the dietary intervention were mediated by diet composition changes or caloric restriction. Finally, we did not record feeding times; although, it would have been interesting to take into account the effect of circadian rhythms. Despite these limitations, we consider our results to be basically valid, thoroughly showing the effects of diet, age, and pharmacological treatment on lipid levels in liver and adipose tissue.

## 5. Conclusions

Our data show that age affects changes in the adipocyte lipidome and that HFHSD diets promote alterations in hepatic lipidome leading to the NASH phenotype. This is independent of metformin administration. As such, we suggest that metformin ought not to be considered a preventive strategy for attenuating NAFLD development. However, using dietary interventions that mediate caloric reduction intake in combination with metformin could be an efficient treatment, and a good strategy for inducing greater dietary intervention effects.

## Data Availability

Data associated with this article can be obtained from the corresponding authors upon reasonable request.

## Supplementary Materials

The following supporting information can be downloaded at https://www.mdpi.com/article/10.3390/biom12121787/s1, Table S1: Nutritional composition of chow diet and high fat-high sucrose diet. Figure S1: Study design scheme; Figure S2: Effects of metformin on biochemical variables; Figure S3: Metformin does not improve glucose tolerance test in obese mice; Figure S4: High-fat high-sucrose diet and age adversely affect the lipoprotein profile; Figure S5: Effects of metformin on lipoproteins profiles; Figure S6: Effects of metformin on lipoprotein particle size; Figure S7: Effects of metformin on hepatic histology; Figure S8: Hepatic lipid signature according to diet, age, and metformin treatment; Figure S9: Adipocyte lipid signature according to metformin treatment; Figure S10: Adipocyte lipid signature according to dietary treatment; Figure S11: Lipid class distribution in epididymal adipocytes; Figure S12: Lipid class distribution in retroperitoneal adipocytes; Figure S13: Lipid class distribution in inguinal adipocytes; Figure S14: Lipid class distribution in anterior adipocytes; Figure S15: Age, diet, and metformin effects in adipocyte lipid categories; Figure S16: Caloric restriction increased the lipid unsaturation in adipocytes.
