# Supplementary material for: Combining Dietary Intervention with Metformin Treatment Enhances Non-Alcoholic Steatohepatitis Remission in Mice Fed a High-Fat High-Sucrose Diet
Source: Biomolecules. 2022 Nov 30;12(12):1787. doi: 10.3390/biom12121787 (PMC9775246; doi:10.3390/biom12121787)
Supplement: Supplementary file 1 [file biomolecules-12-01787-s001.zip › biomolecules-1909627-supplementary.pdf]

Combining dietary intervention with metformin treatment enhances NAFLD remission in mice fed a high-fat high-sucrose diet.

Gerard Baiges-Gaya, Elisabet Rodríguez-Tomàs, Helena Castañé, Andrea Jiménez-Franco, Núria Amigó, Jordi Camps, Jorge Joven

Table of contents

Table S1……………………………………………………………………………….2
Fig. S1…………………………………………………………………………………..3
Fig. S2…………………………………………………………………………………..4
Fig. S3…………………………………………………………………………………..5
Fig. S4…………………………………………………………………………………..6
Fig. S5…………………………………………………………………………………..8
Fig. S6…………………………………………………………………………………10
Fig. S7…………………………………………………………………………………11
Fig. S8…………………………………………………………………………………12
Fig. S9…………………………………………………………………………………13
Fig. S10………………………………………………………………………………..14
Fig. S11………………………………………………………………………………..16
Fig. S12………………………………………………………………………………..17
Fig. S13………………………………………………………………………………..18
Fig. S14………………………………………………………………………………..19
Fig. S15………………………………………………………………………………..20
Fig. S16………………………………………………………………………………..21

	CD	HFHSD
Crude protein (%)	14.3	17.3
Crude fat (%)	4	23.2
Crude fibre (%)	4.1	5.0
Carbohydrate (available) (%)	48	47.6
Crude ash (%)	4.7	ND
Nitrogen free extracts (%)	43.5	ND
Energy density (Kcal/g (Kj/g))	2.9 (12.1)	4.7 (19.66)
Calories from protein (%)	20	14.7
Calories from fat (%)	13	44.6
Calories from carbohydrate (%)	67	40.7
Calcium (%)	0.7	0.62
Phosphorus (%)	0.6	0.33
Sodium (%)	0.1	0.13
Magnesium (%)	0.2	0.06
Potassium (%)	0.6	0.44
Chloride (%)	0.3	0.19
Caprid acid (C10:0) (%)	ND	0.42
Lauric acid (C12:0) (%)	ND	0.65
Myristic acid (C14:0) (%)	ND	2.46
Palmitic acid (C16:0) (%)	0.5	5.72
Palmitoleic acid (C16:1) (%)	ND	0.4
Stearic acid (C18:0) (%)	0.1	2.71
Oleic acid (C18:1) (%)	0.7	6.39
Linoleic acid (C18:2) (%)	2	1.67
Linolenic acid (C18:3) (%)	0.1	0.27
Cholesterol (mg/kg)	ND	576.6
Lysine (%)	0.7	1.37
Methionine (%)	0.4	0.45
Cystine (%)	0.3	0.35
Threonine (%)	0.5	0.74
Tryptophan (%)	0.2	0.2
Arginine (%)	0.8	0.64
Histidine (%)	0.4	0.49
Valine (%)	0.7	1.17
Isoleucine (%)	0.6	0.98
Leucine (%)	1.4	1.56
Phenylalanine (%)	0.7	0.86
Glycine (%)	0.7	0.31
Glutamic acid (%)	2.9	3.55
Aspartic acid (%)	0.9	1.17
Proline (%)	1.2	1.76
Alanine (%)	0.9	0.51
Serine (%)	0.7	0.98
Vitamin A (IU/kg)	17000	14068
Vitamin D_3 (IU/kg)	1200	1900
Vitamin E (IU/kg)	150	143
Vitamin K (as menadione) (mg/Kg)	58	1.4
Thiamin (B_1) (mg/Kg)	64	9.2
Riboflavin (B_2) (mg/Kg)	16	11.4
Pyridoxime (B_6) (mg/Kg)	17	10.9
Cyanocobalamin (B_{12}) (mg/Kg)	0.09	0.05
Nicotinic acid (mg/Kg)	84	57
Pantothenic acid (mg/Kg)	76	27.8
Folic acid (mg/Kg)	5	3.8
Biotin (mg/Kg)	0.57	0.38
Choline-Chloride (mg/Kg)	1030	1337
Iron (mg/Kg)	175	45.4
Manganese (mg/Kg)	100	12.9
Zinc (mg/Kg)	70	49.6
Copper (mg/Kg)	15	7.6
Iodine (mg/Kg)	6	0.23
Selenium (mg/Kg)	0.23	0.18

Table S1. Nutritional composition of chow diet (CD; TD.2014, Harlan Laboratories Inc., Madison, WI, USA) and high fat-high sucrose diet (HFHSD; TD.08811, Harlan Laboratories Inc., Madison, WI, USA)

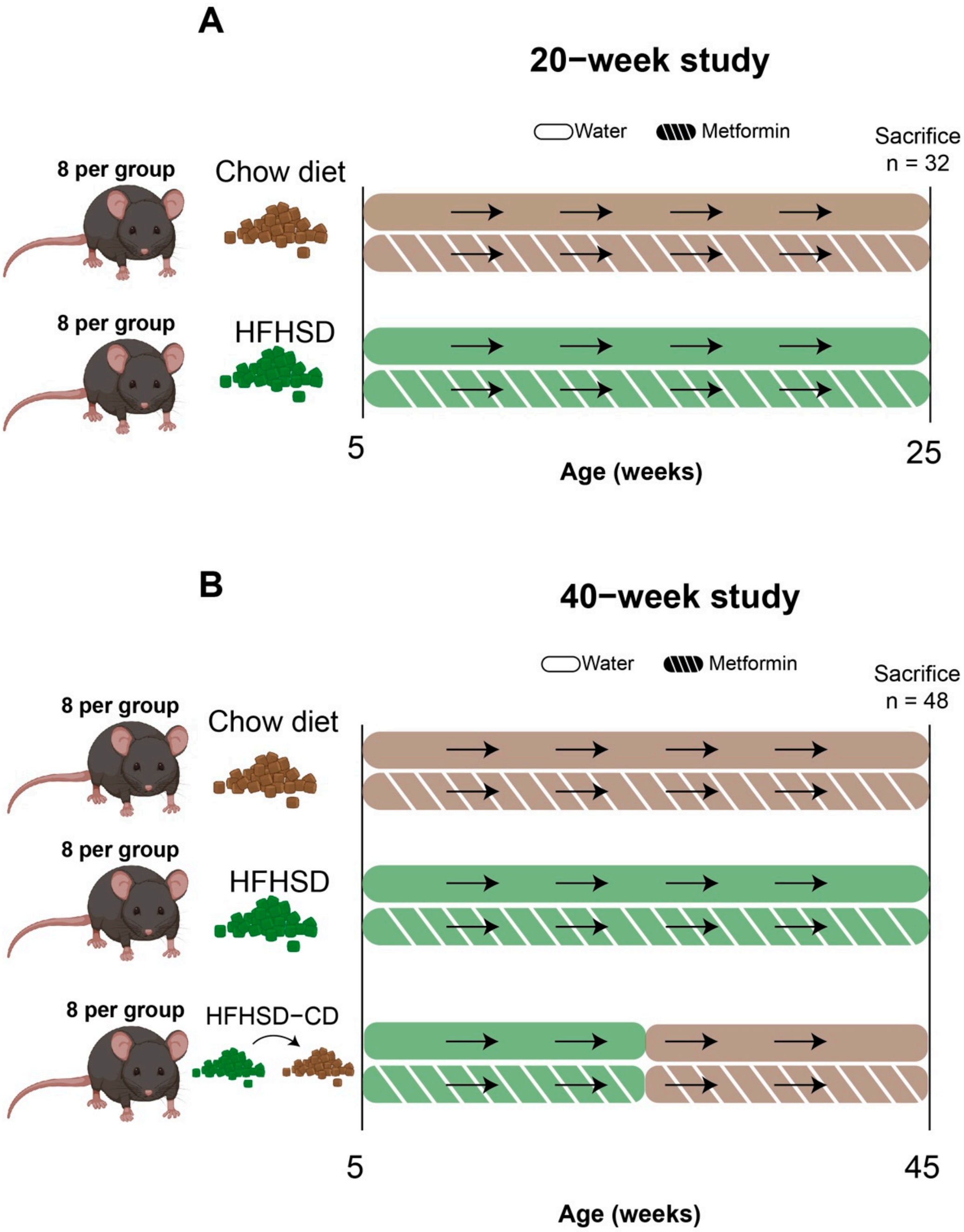

Fig. S1. Study design scheme
(**A**) Scheme of 20-week and (**B**) 40-week study design. HFHSD, high-fat high-sucrose diet.

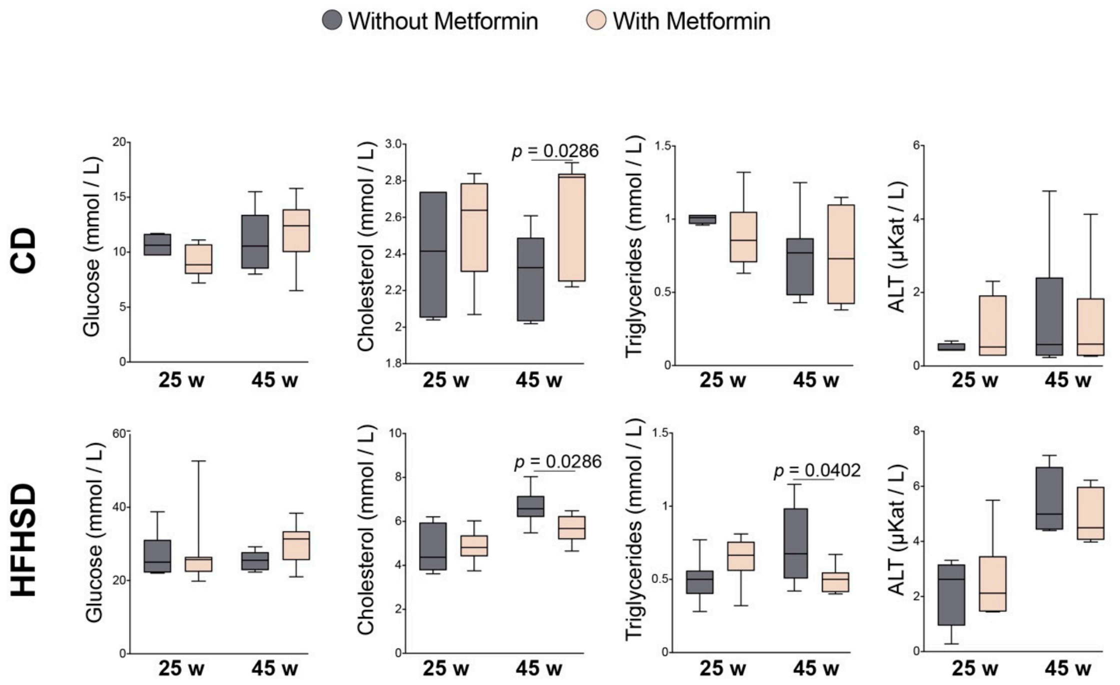

Fig. S2. Effects of metformin on biochemical variables
Glucose, cholesterol, and triglyceride concentrations, and alanine aminotransferase (ALT) activities in mice fed with chow or high-fat high-sucrose diets (CD and HFHSD, respectively) (*n* = 8/group). Data are presented as means, maximum and minimum. p values < 0.05 are considered significant. (Wilcoxon-rank sum test).

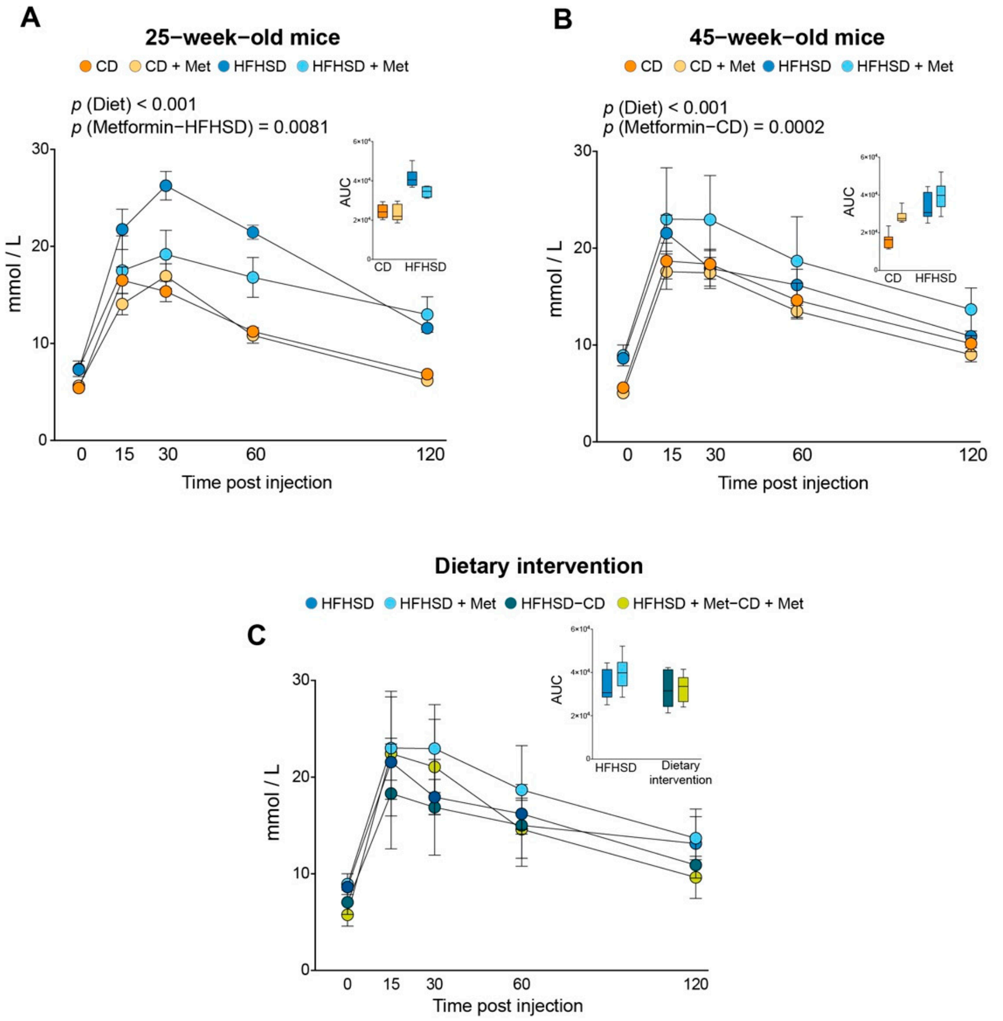

Fig. S3. Metformin does not improve glucose tolerance test in obese mice
(**A**) Glucose tolerance test curves and areas under the curve (AUC) of 25-week-old mice, and (**B**) 45-week-old mice. (**C**) Effect of dietary intervention, with or without metformin treatment, on glucose tolerance test (*n* = 8/group). Data are shown as means and SD. *p* values < 0.05 are considered significant. (Wilcoxon-rank sum test). CD: chow diet; HFHSD: high-fat high-sucrose diet; Met: metformin.

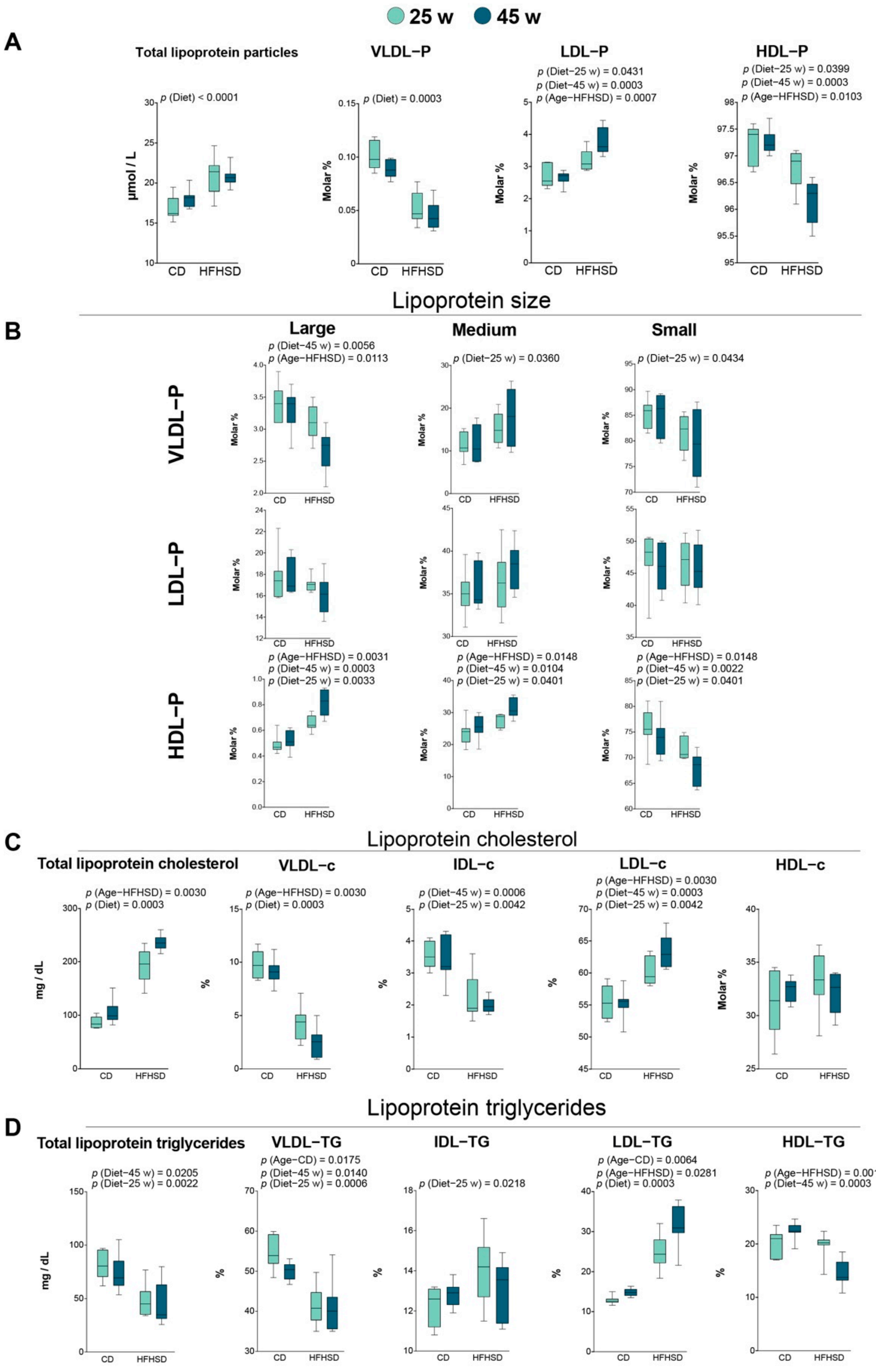

Fig. S4. High-fat high-sucrose diet and age adversely affect the lipoprotein profile
(**A**)Total concentration of lipoprotein particles and VLDL-p, LDL-p, and HDL-p molar abundance (expressed as a percentage of total measured lipoprotein particles) (*n* = 4/group). (**B**) The molar abundance of lipoprotein size (expressed as a percentage of total measured VLDL-p, LDL-p or HDL-p) (*n* = 4/group). (**C**) Total lipoprotein cholesterol concentration and abundance of VLDL-c, IDL-c,

LDL-c and HDL-c (expressed as a percentage of total measured lipoprotein cholesterol) (n = 4/group). (**D**)Total lipoprotein triglyceride concentrations and abundance of VLDL-TG, IDL-TG, LDL-TG and HDL-TG (expressed as a percentage of total measured lipoprotein triglycerides) (*n* = 4/group). Data are shown as means, maximum and minimum. p values < 0.05 are considered significant. (Wilcoxon-rank sum test). CD: chow diet; HDL: High-density lipoproteins; HFHSD: high-fat high-sucrose diet; IDL: intermediate-density lipoproteins; LDL: low-density lipoproteins; p: particles; TG: triglycerides; VLDL: very low-density lipoproteins; w: weeks.

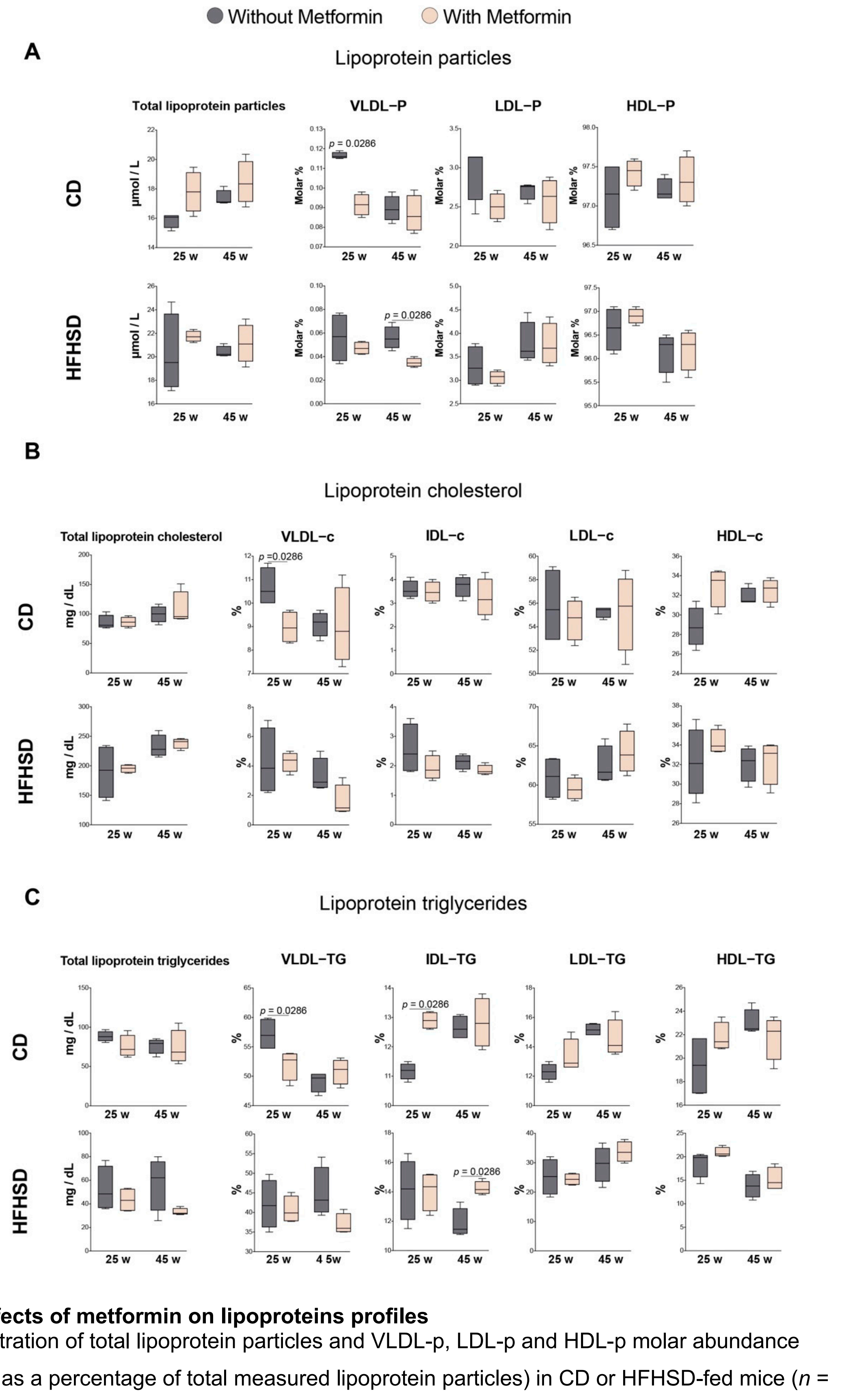

Fig. S5. Effects of metformin on lipoproteins profiles
(**A**) Concentration of total lipoprotein particles and VLDL-p, LDL-p and HDL-p molar abundance (expressed as a percentage of total measured lipoprotein particles) in CD or HFHSD-fed mice (n = 4/group). (**B**) Total lipoprotein cholesterol concentration and abundance of VLDL-c, IDL-c, LDL-c and HDL-c (expressed as a percentage of total measured lipoprotein cholesterol) in CD or HFHSD-fed

mice (*n* = 4/group). (**C**)Total lipoprotein triglyceride concentrations and abundance of VLDL-TG, IDL-TG, LDL-TG and HDL-TG (expressed as a percentage of total measured lipoprotein triglycerides) in CD or HFHSD-fed mice (*n* = 4/group). Data are shown as means, maximum and minimum. *p* values < 0.05 are considered significant. (Wilcoxon-rank sum test). CD: chow diet; HDL: high-density lipoproteins; HFHSD: high-fat high-sucrose diet; IDL: intermediate-density lipoproteins; LDL: low-density lipoproteins; p: particles; TG: triglycerides; VLDL: very low-density lipoproteins; w: weeks.

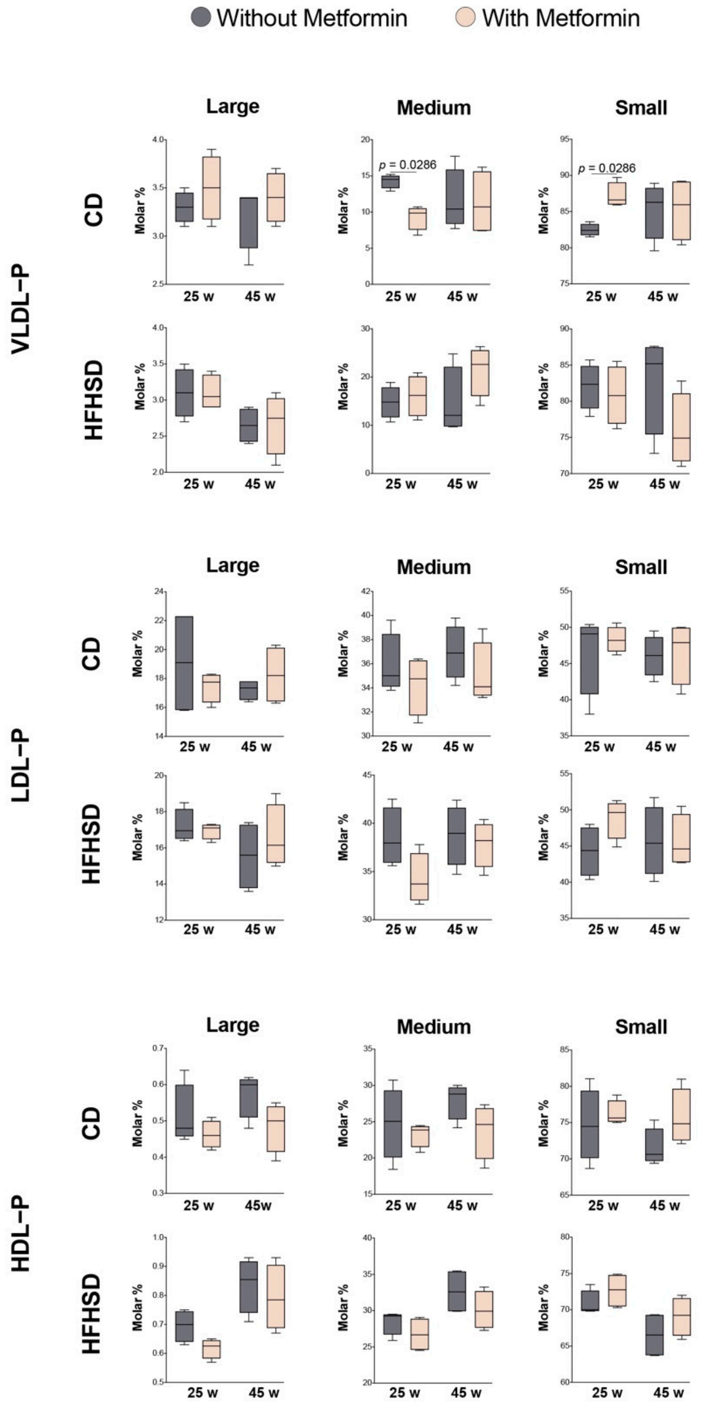

Fig. S6. Effects of metformin on lipoprotein particle size
(**A-C**) The molar abundance of lipoprotein size (expressed as a percentage of total measured VLDL-p, LDL-p or HDL-p) in CD or HFHSD-fed mice (*n* = 4/group). Data are shown as means, maximum and minimum. *p* values < 0.05 are considered significant. (Wilcoxon-rank sum test). CD: chow diet; HDL: high-density lipoproteins; HFHSD: high-fat high-sucrose diet; IDL: intermediate-density lipoproteins; LDL: low-density lipoproteins; p: particles; TG: triglycerides; VLDL: very low-density lipoproteins; w: weeks.

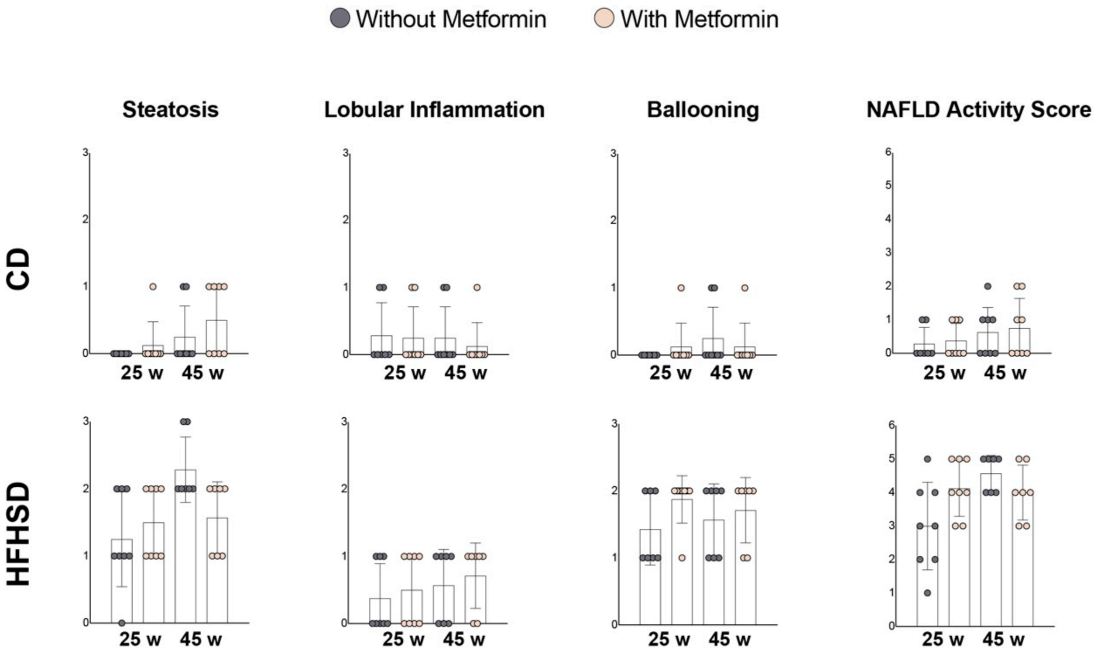

Fig. S7. Effects of metformin on hepatic histology
Hepatic steatosis, lobular inflammation, ballooning, and NAFLD activity scores in CD or HFHSD-fed mice with or without metformin treatment (n = 8/group). Data shown as means and SD. p values < 0.05 are considered significant. (Wilcoxon-rank sum test). CD: chow diet; HFHSD: high-fat high sucrose diet; w: weeks.

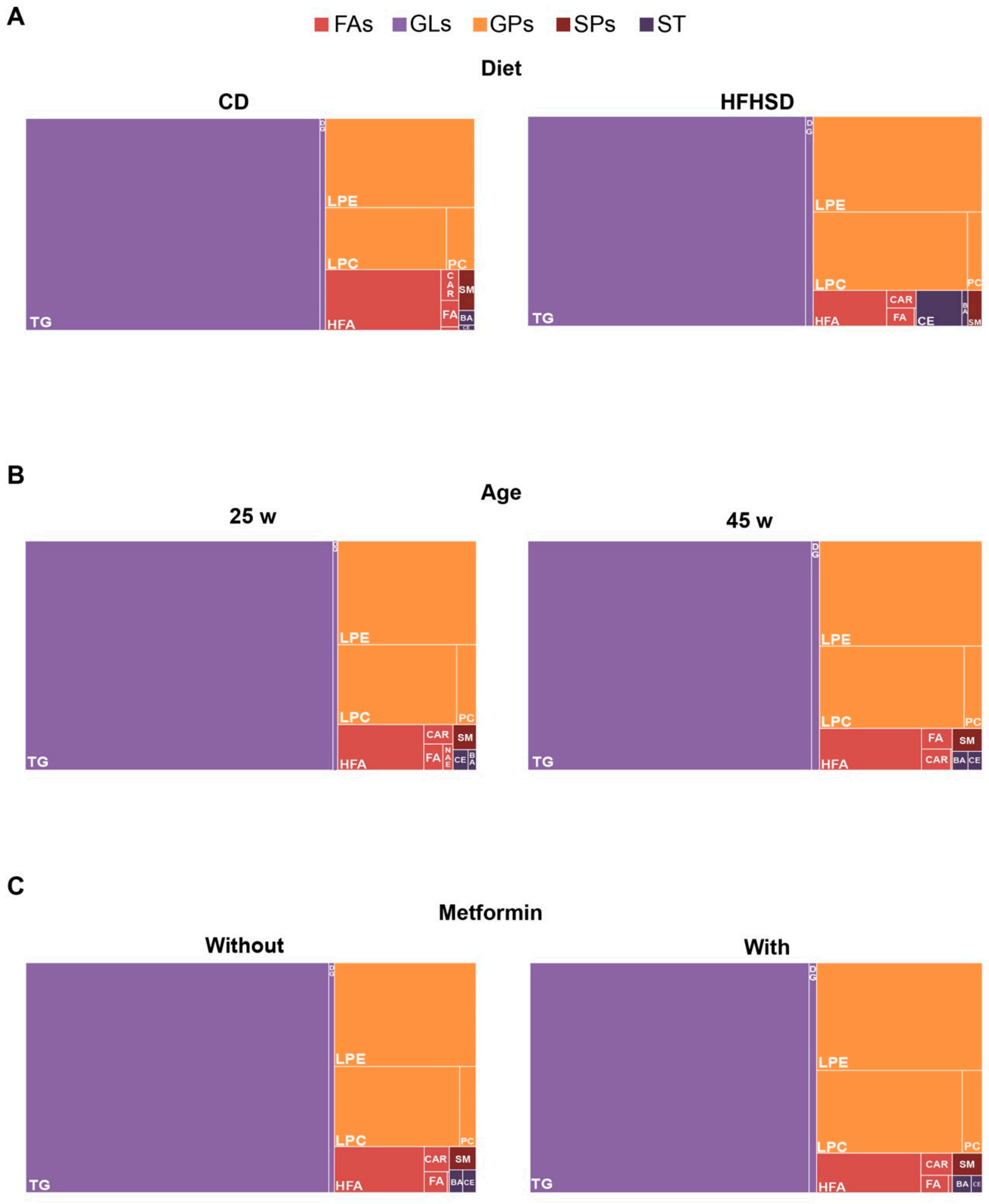

Fig. S8. Hepatic lipid signature according to diet, age, and metformin treatment
Treemaps show the relative distribution of main lipid categories and classes (*n* = 4/group). These relative distributions serve as a comparative metric of hepatic lipidome between (**A**) dietary treatments, (**B**) age, (**C**) metformin treatment. FAs, fatty acyls; GLs, glycerolipids; GPs, glycerophospholipids; BA: bile acids; CAR: carnitines; CE: cholesterol esters; DG, diglycerides; FA: fatty acids; HFA: hydroxy fatty acids; LPC: lysophosphatidylcholine; LPE: lysophosphatidylethanolamine; PC: phosphatidylcholines; SM: sphingomyelins; SPs: sphingophospholipids, ST: sterol lipids; w: weeks.

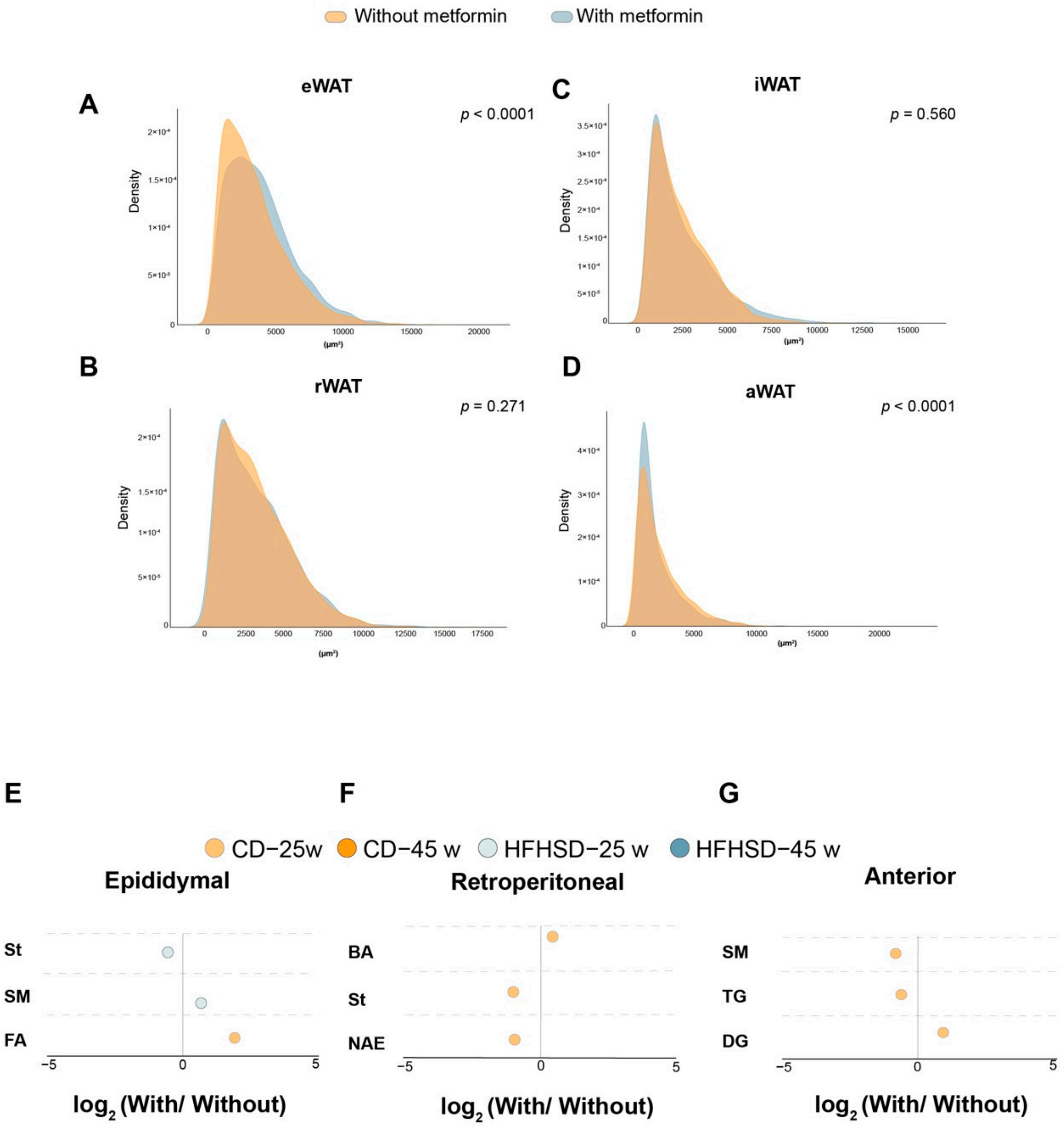

Fig. S9. Adipocyte lipid signature according to metformin treatment

Density plots show the relative frequency of histological adipocyte size from (**A**) epididymal (eWAT), (**B**) retroperitoneal (rWAT), (**C**) inguinal (iWAT) and (**D**) anterior (aWAT) white adipose tissues. The metformin treatment highlighted in sky-orange (mice without metformin) and sky-blue (mice with metformin) (*n* = 8/group). Representation of significant lipid classes, showing the mean $\log_2$ (with metformin / without metformin) in (E) epididymal, (**F**) retroperitoneal, and (**G**) anterior adipocytes (*n* = 4/group). Groups of mice represented in sky-orange (25-week-old mice with CD) or orange (45-week-old mice with CD) (*n* = 4/group) and sky-blue (25-week-old mice with HFHSD) or blue (45-week-old mice with HFHSD) dots (*n* = 4/group). *p* values < 0.05 are considered significant. (Wilcoxon-rank sum test). BA: bile acids; CD: chow diet; DG: diglycerides; FA: fatty acids; HFHSD: high-fat high-sucrose diet; NAE: N-acyl-ethanolamine's; SM: sphingomyelins; St: steroid hormones; TG: triglycerides; w: weeks.

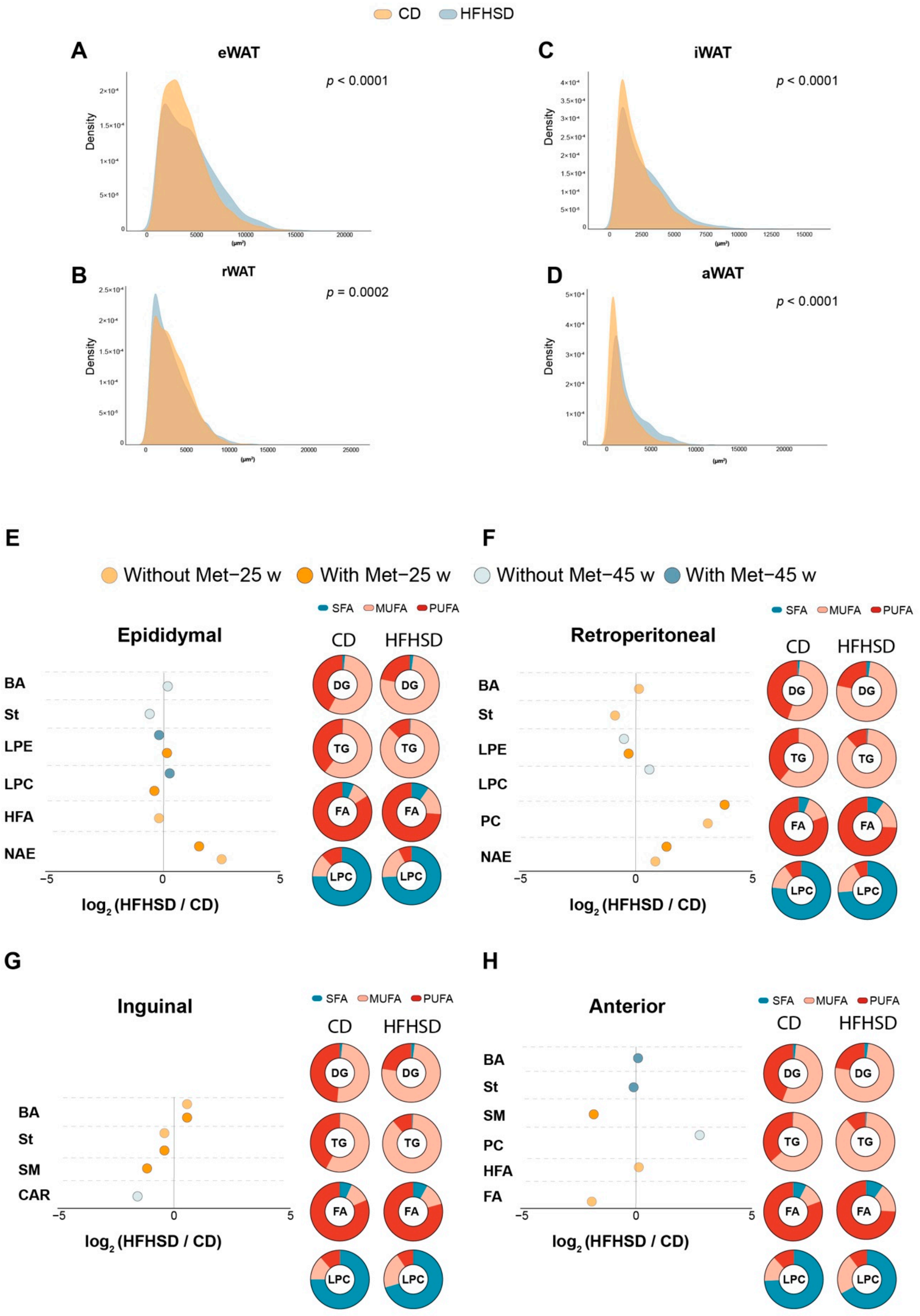
CD
HFHSD
A
eWAT
p < 0.0001
Density
(μm²)
B
rWAT
p = 0.0002
Density
(μm²)
C
iWAT
p < 0.0001
Density
(μm²)
D
aWAT
p < 0.0001
Density
(μm²)
E
F
Without Met−25 w
With Met−25 w
Without Met−45 w
With Met−45 w
SFA
MUFA
PUFA
Epididymal
CD
HFHSD
BA
St
LPE
LPC
HFA
NAE
log2 (HFHSD / CD)
DG
TG
FA
LPC
Retroperitoneal
BA
St
LPE
LPC
PC
NAE
log2 (HFHSD / CD)
G
Inguinal
BA
St
SM
CAR
log2 (HFHSD / CD)
H
Anterior
BA
St
SM
PC
HFA
FA
log2 (HFHSD / CD)

Fig. S10. Adipocyte lipid signature according to dietary treatment
Density plots show the relative frequency of histological adipocyte size from (**A**) epididymal (eWAT), (**B**) retroperitoneal (rWAT), (**C**) inguinal (iWAT) and (**D**) anterior (aWAT) white adipose tissues. Diets of mice colored in sky-orange (CD-fed mice) and sky-blue (HFHSD-fed mice) (n = 8/group). Representation of significant lipid classes showing the mean $\log_2$ (HFHSD-fed mice / CD-fed mice) and the unsaturation changes in (**E**) epididymal (top left), (**F**) retroperitoneal (top right), (**G**) inguinal (bottom left) and (**H**) anterior (bottom right) adipocytes. Groups of mice represented in sky-orange (without metformin) or orange (with metformin) dots in 25-week-old mice (n = 4/group) and sky-blue (without metformin) or blue (with metformin) dots in 45-week-old mice (n = 4/group). p values < 0.05 are considered significant. (Wilcoxon-rank sum test). BA: bile acids; CAR: carnitines; CD: chow diet; DG: diglyceride; FA: fatty acid; HFA: hydroxy fatty acids; HFHSD: high-fat high-sucrose diet; LPC: lysophosphatidylcholines; LPE: lysophosphatidylethanolamines; Met: metformin; MUFA: monounsaturated fatty acids; NAE: N-acyl-ethanolamine's; PC: phosphatidylcholines; PUFA: polyunsaturated fatty acids; SM: sphingomyelins; SFA: saturated fatty acids; ST: steroid hormones; TG: triglycerides; w: weeks.

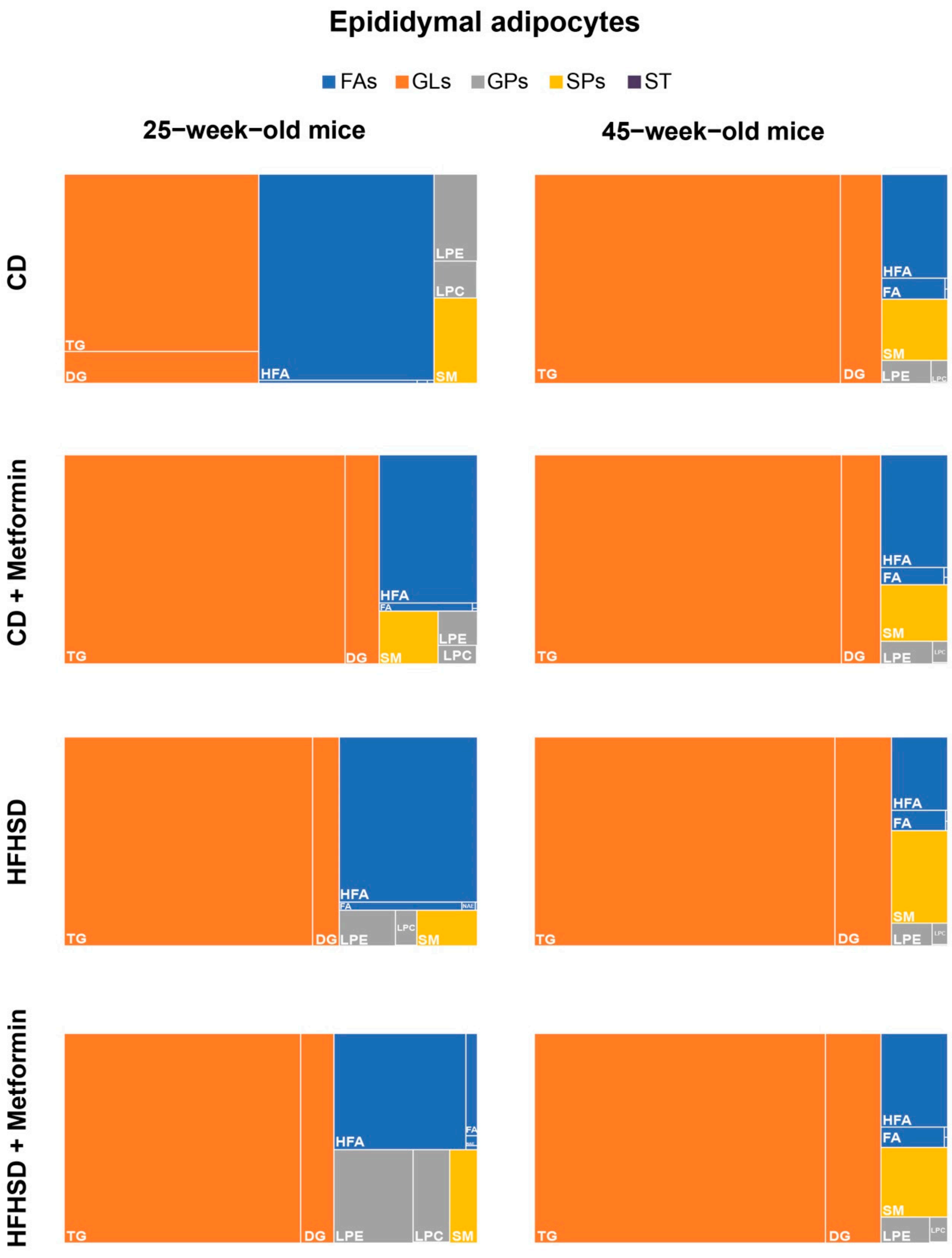

Fig. S11. Lipid class distribution in epididymal adipocytes
Treemaps show the relative distribution of main lipid categories and classes in epididymal adipocytes (*n* = 4/group). These relative distributions serve as a comparative metric of epididymal lipidome between dietary treatments, age, and metformin treatment. BA: bile acids; CAR: carnitine; CD: chow diet; DG: diglycerides; FA: fatty acids; FAs: fatty acyls; GLs: glycerolipids; GPs: glycerophospholipids, HFA: hydroxy fatty acids; HFHSD: high-fat high sucrose diet; LPC: lysophosphatidylcholines; LPE: lysophosphatidylethanolamines; NAE: N-acyl-ethanolamine’s; PC: phosphatidylcholines; SM: sphingomyelins; SPs: sphingophospholipids; St: steroid hormones; ST: sterol lipids, TG: triglycerides.

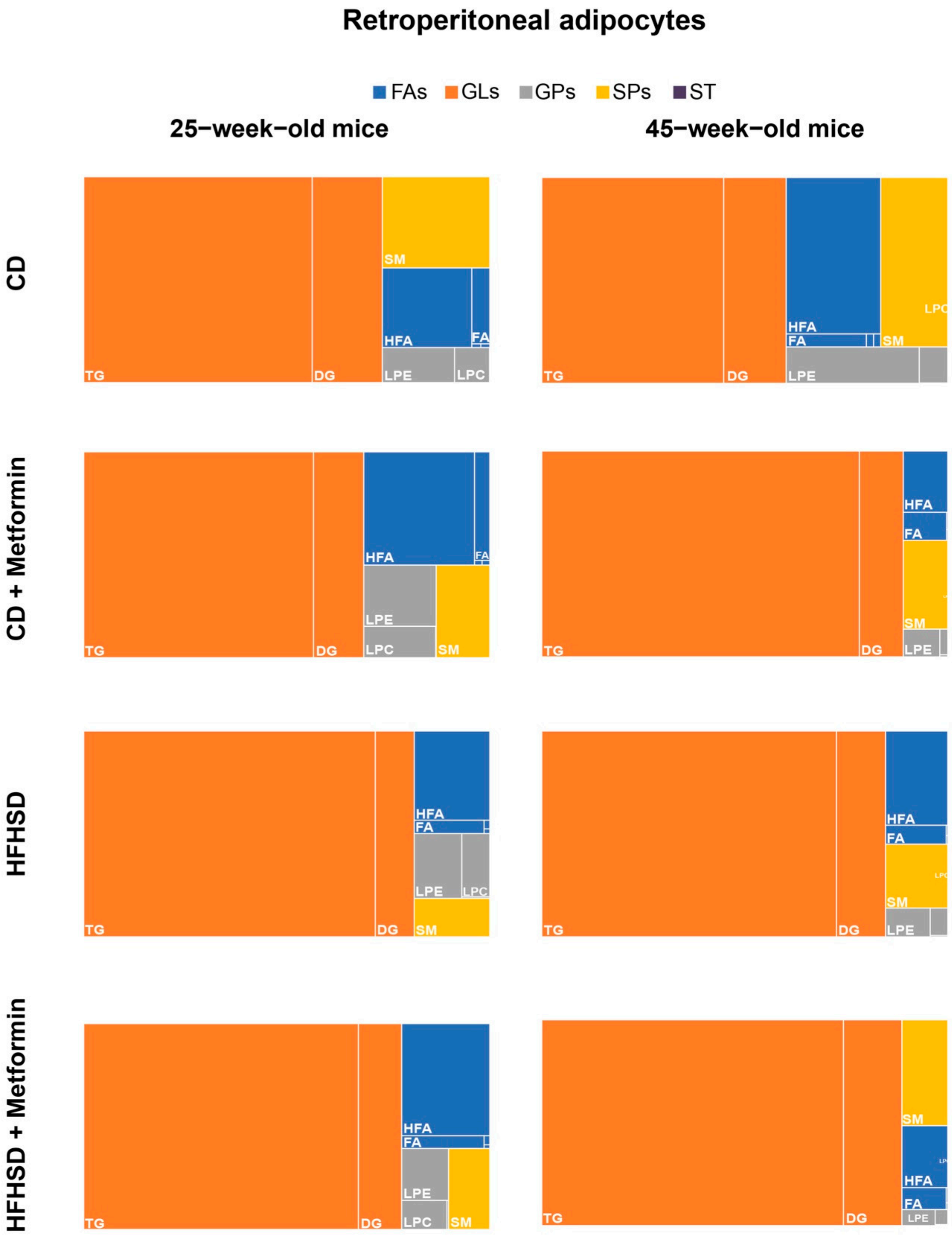

Fig. S12. Lipid class distribution in retroperitoneal adipocytes
Treemaps show the relative distribution of main lipid categories and classes in retroperitoneal adipocytes (*n* = 4/group). These relative distributions serve as a comparative metric of retroperitoneal lipidome between dietary treatments, age, and metformin treatment. BA: bile acids; CAR: carnitine; CD: chow diet; DG: diglycerides; FA: fatty acids; FAs: fatty acyls; GLs: glycerolipids; GPs: glycerophospholipids, HFA: hydroxy fatty acids; HFHSD: high-fat high sucrose diet; LPC: lysophosphatidylcholines; LPE: lysophosphatidylethanolamines; NAE: N-acyl-ethanolamine's; PC: phosphatidylcholines; SM: sphingomyelins; SPs: sphingophospholipids; St: steroid hormones; ST: sterol lipids, TG: triglycerides.

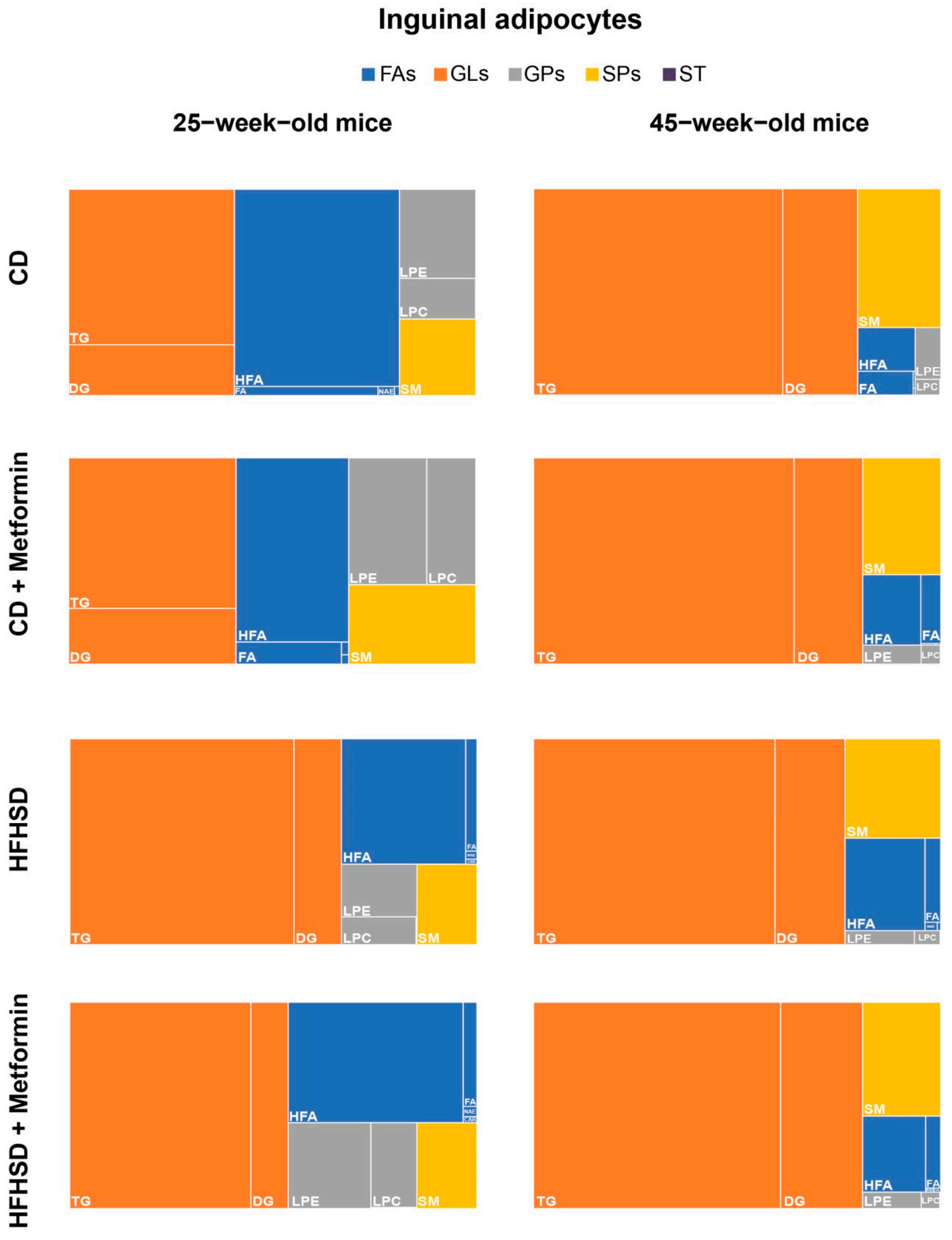

Fig. S13. Lipid class distribution in inguinal adipocytes
Treemaps show the relative distribution of main lipid categories and classes in inguinal adipocytes (*n* = 4/group). These relative distributions serve as a comparative metric of inguinal lipidome between dietary treatments, age, and metformin treatment. BA: bile acids; CAR: carnitine; CD: chow diet; DG: diglycerides; FA: fatty acids; FAs: fatty acyls; GLs: glycerolipids; GPs: glycerophospholipids, HFA: hydroxy fatty acids; HFHSD: high-fat high sucrose diet; LPC: lysophosphatidylcholines; LPE: lysophosphatidylethanolamines; NAE: N-acyl-ethanolamine's; PC: phosphatidylcholines; SM: sphingomyelins; SPs: sphingophospholipids; St: steroid hormones; ST: sterol lipids, TG: triglycerides.

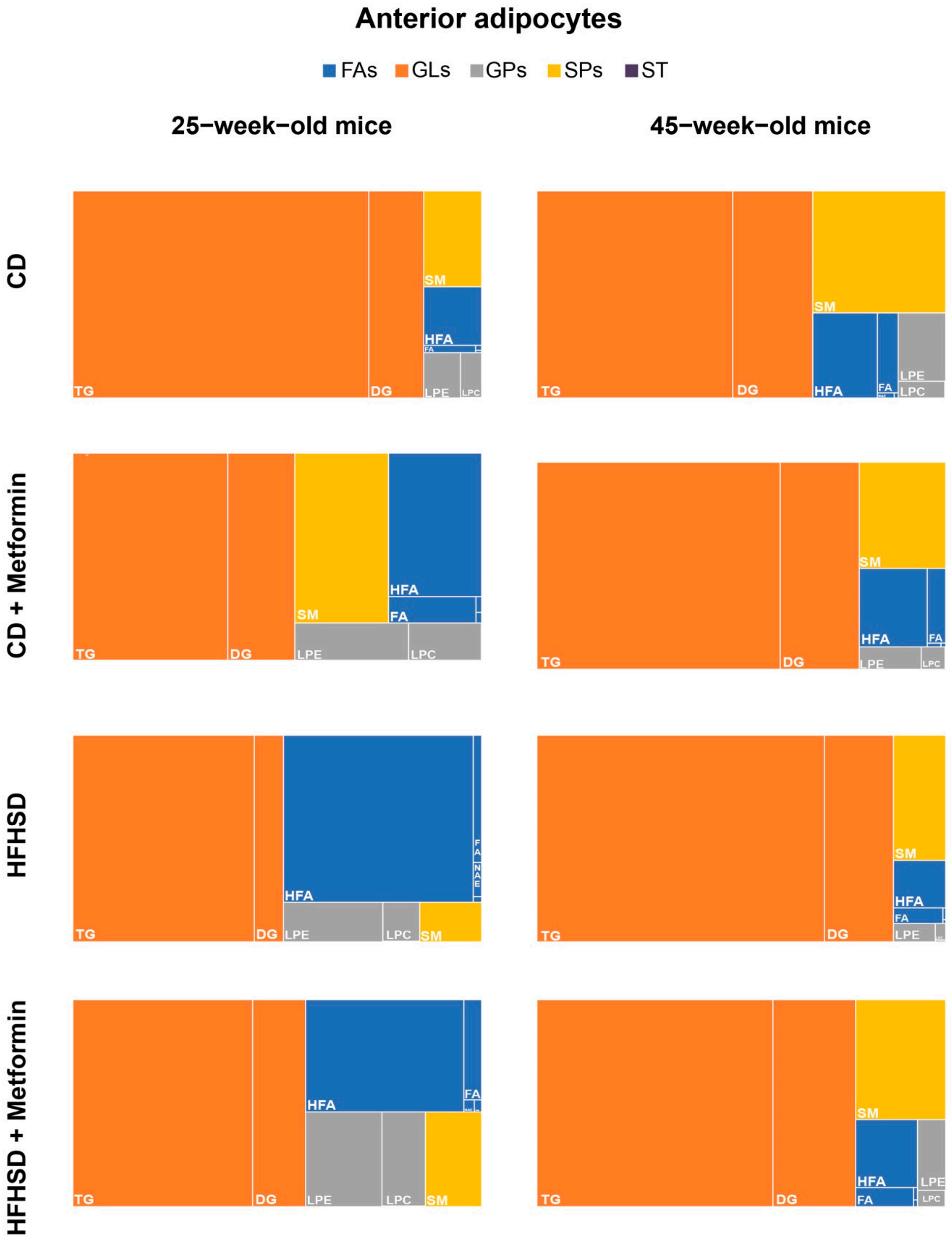

Fig. S14. Lipid class distribution in anterior adipocytes
Treemaps show the relative distribution of main lipid categories and classes in anterior adipocytes (*n* = 4/group). These relative distributions serve as a comparative metric of anterior lipidome between dietary treatments, age, and metformin treatment. BA: bile acids; CAR: carnitine; CD: chow diet; DG: diglycerides; FA: fatty acids; FAs: fatty acyls; GLs: glycerolipids; GPs: glycerophospholipids, HFA: hydroxy fatty acids; HFHSD: high-fat high sucrose diet; LPC: lysophosphatidylcholines; LPE: lysophosphatidylethanolamines; NAE: N-acyl-ethanolamine’s; PC: phosphatidylcholines; SM: sphingomyelins; SPs: sphingophospholipids; St: steroid hormones; ST: sterol lipids, TG: triglycerides.

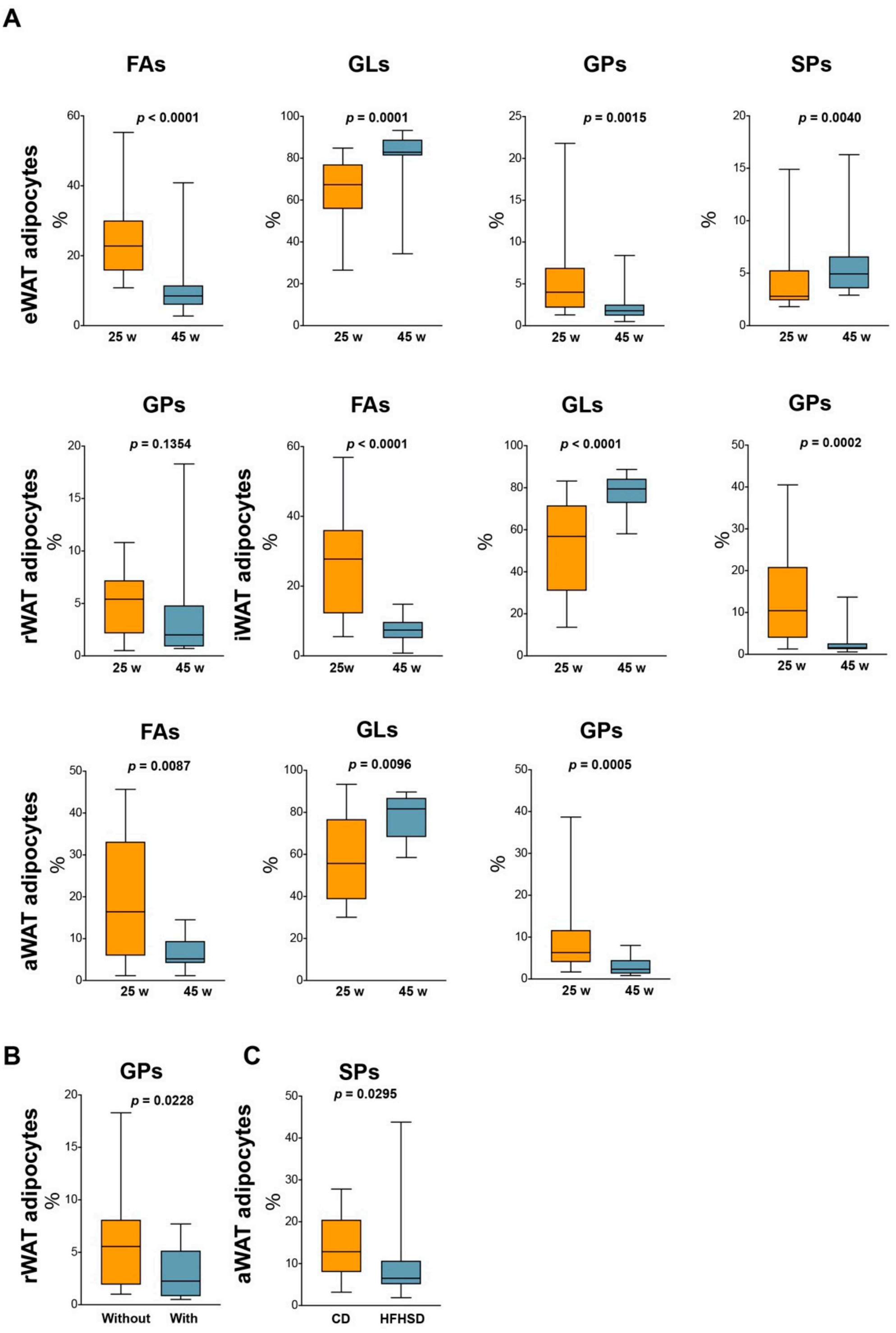

Fig. S15. Age, diet and metformin effects in adipocyte lipid categories

(**A**) Box plots show the lipid category alterations in visceral and subcutaneous adipocytes according to age, (**B**) metformin and (**C**) diet (*n* = 4/group). Data are presented as means, maximum and minimum. *p* values < 0.05 are considered significant. (Wilcoxon-rank sum test). aWAT: anterior white adipose tissue; CD: show diet; eWAT: epididymal white adipose tissue; FAs: fatty acyls; GLs: glycerolipids; GPs: glycerophospholipids; HFHSD: high-fat high sucrose diet; iWAT: inguinal white adipose tissue; rWAT: retroperitoneal white adipose tissue; SPs: sphingophospholipids, ST: sterol lipids.

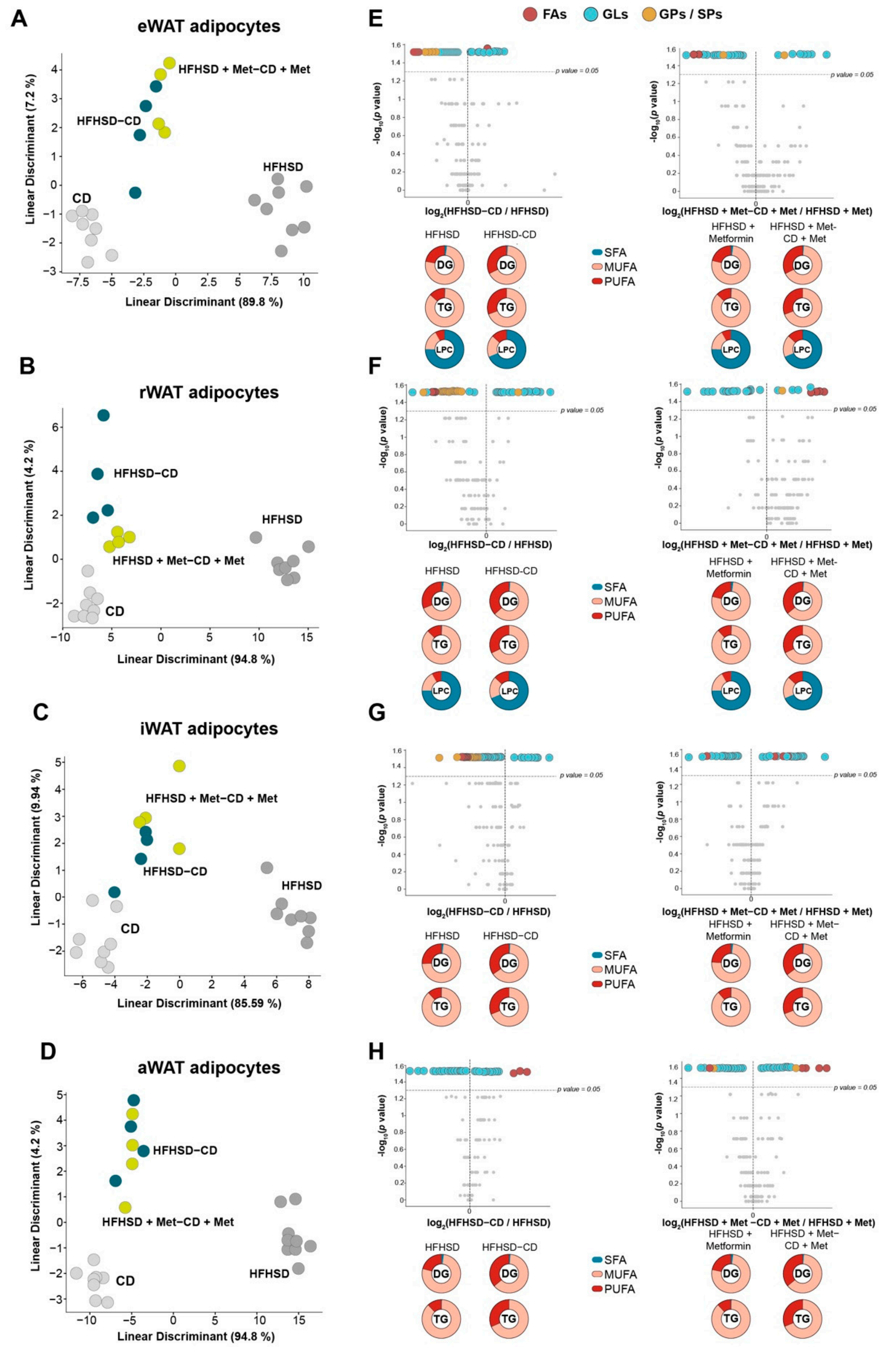

Fig. S16. Dietary intervention increased the lipid unsaturation in adipocytes

Lipid signature analysis between dietary intervention with or without metformin in obese mice in (**A**) epididymal (eWAT), (**C**) retroperitoneal (rWAT), (**E**) inguinal (iWAT), (**G**) anterior (aWAT) adipocytes (*n* = 4/group). (**B**) Volcano plots of the effect of dietary intervention with or without metformin

administration in (**B**-**D**) visceral and (**F**-**H**) subcutaneous adipocytes (*n* = 4/group). Significant lipid species are colored according to the lipid category. Levels of significance below 0.05 were considered significant (Wilcoxon-rank sum test).CD: chow diet; DG: diglycerides; FAs: fatty acyls; GLs: glycerolipids; GPs: glycerophospholipids; HFHSD: high-fat high-sucrose diet LPC: lysophosphatidylcholines; Met: metformin; MUFA: monounsaturated fatty acids; PUFA: polyunsaturated fatty acids, SFA: saturated fatty acids; SPs: sphingophospholipids; TG: triglycerides.
